# Hypothalamic Astrocytes Exhibit Glycolytic Features Making Them Prone for Glucose Sensing

**DOI:** 10.1002/glia.70066

**Published:** 2025-07-24

**Authors:** Sarah Geller, Nadège Zanou, Sylviane Lagarrigue, Tamara Zehnder, Cathy Gouelle, Tania Santoro, Cendrine Repond, Paola Bezzi, Francesca Amati, Anne‐Karine Bouzier‐Sore, Ariane Sharif, Luc Pellerin

**Affiliations:** ^1^ Department of Physiology University of Lausanne Lausanne Switzerland; ^2^ Center for Integrative Genomics University of Lausanne Lausanne Switzerland; ^3^ Institute of Sports Sciences University of Lausanne Lausanne Switzerland; ^4^ Department of Biomedical Sciences University of Lausanne Lausanne Switzerland; ^5^ Department of Fundamental Neuroscience University of Lausanne Lausanne Switzerland; ^6^ Department of Oncology, Centre Hospitalier Universitaire Vaudois and Ludwig Institute for Cancer Reaserach Lausanne Switzerland; ^7^ Department of Physiology and Pharmacology Sapienza University of Rome Rome Italy; ^8^ Service of Endocrinology, Diabetes and Metabolism Lausanne University Hospital and University of Lausanne Lausanne Switzerland; ^9^ Centre de Résonance Magnétique Des Systèmes Biologiques, UMR5536 CNRS Université de Bordeaux, CNRS Bordeaux France; ^10^ Laboratory of Development and Plasticity of the Neuroendocrine Brain, Lille Neuroscience & Cognition, UMR‐S 1172, FHU 1000 Days for Health University of Lille (Univ. Lille), Inserm, CHU Lille Lille France; ^11^ Ischémie Reperfusion, Métabolisme et Inflammation Stérile en Transplantation, Unité 1313 INSERM Université de Poitiers et CHU de Poitiers Poitiers France

**Keywords:** astrocytes, glucose, hypothalamus, lactate, Mct1, Mct4, Pkm2

## Abstract

In the hypothalamus, detection of energy substrates such as glucose is essential to regulate food intake and peripheral energy homeostasis. Metabolic interactions between astrocytes and neurons via lactate exchange have been proposed as a hypothalamic glucose‐sensing mechanism, but the molecular basis remains uncertain. Mouse hypothalamic astrocytes in vitro were found to exhibit a stronger glycolytic phenotype in basal conditions than cortical astrocytes. It was associated with higher protein expression levels of the Pyruvate Kinase Isoform M2 (Pkm2) and its more prominent nuclear localization. In parallel, hypothalamic astrocytes also expressed higher levels of the monocarboxylate transporter *Slc16a3* (Mct4), which were dependent on Pkm2 expression. The stronger Mct4 expression in hypothalamic versus cortical astrocytes is an intrinsic characteristic, as it was also present after their direct isolation from adult mouse tissue. The high lactate release capacity of hypothalamic astrocytes was demonstrated to depend on the expression of Mct4, but not Mct1. Unlike cortical astrocytes, hypothalamic astrocytes in culture do not respond to glutamate with enhanced glycolysis, but instead, they modulate their lactate production according to glucose concentrations in an AMPK‐dependent manner, an effect observed in both mouse and human hypothalamic astrocytes in vitro. Our study shows that hypothalamic and cortical astrocytes are geared to have distinct glycolytic responses to glucose and glutamate, respectively. These results reveal a metabolic specialization of astrocytes in order to fulfill distinct area‐specific functions: glucose‐sensing in the hypothalamus versus activity‐dependent neuronal energetic supply in cortical regions.

## Main Points

- Hypothalamic astrocytes versus cortical astrocytes:
  - are more glycolytic due to high Pkm2, Glut1 and Mct4 expression
  - do not exhibit glutamate‐induced glycolysis
  - detect glucose concentration variations via an AMPK sensor with altered lactate production.

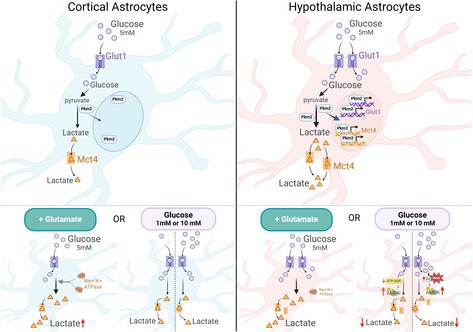

## 1 Introduction

Glycemia regulation is a critical function to ensure proper energy substrate supply to various organs and tissues (including the brain) under various conditions (Sohn and Ho 2020). It depends on the coordinated action of the central nervous system with peripheral organs, as well as the initiation of adapted behaviors such as food intake (Mirzadeh et al. 2022). In order to maintain glucose homeostasis, blood glucose levels must be monitored continuously by the central nervous system. This important detection role is performed within the hypothalamus. The cellular and molecular mechanisms explaining this key hypothalamic property have been the subject of intense investigations in the last decades (Thorens 2024).

A major progress was made with the description of specific neuronal populations within some nuclei of the hypothalamus exhibiting glucose‐sensing properties (Oomura et al. 1969). Indeed, both glucose‐excited and glucose‐inhibited neurons have been identified and proposed to account for hypothalamic glucose sensing (Routh et al. 2014). Nonetheless, it was later found that these neurons could also respond to other energy substrates such as lactate (Mobbs et al. 2001). As lactate can be produced and released locally by glial cells, this observation led to the hypothesis that either astrocytes (Leloup et al. 2016) or tanycytes (Elizondo‐Vega et al. 2015; Lhomme et al. 2021) could contribute to hypothalamic glucose sensing.

Indeed, astrocytes from several brain regions exhibit a high glycolytic capacity (Bouzier‐Sore and Pellerin 2013). Moreover, the same astrocytes can respond to various neuronal, hormonal, or inflammatory signals and upregulate their glycolytic metabolism, leading to higher lactate production, either acutely or for a longer period (Barros et al. 2023; Pellerin et al. 1997; Véga et al. 2002; Zheng et al. 2021). Intriguingly, very little is known about the metabolic profile of hypothalamic astrocytes and whether they can respond to extracellular signals or changes in metabolite levels. In this study, we compared the metabolic properties of hypothalamic astrocytes with those of cortical astrocytes, which have been previously well characterized. It was observed that hypothalamic astrocytes exhibit a stronger glycolytic profile than cortical astrocytes. This is due to higher expression levels of the Pkm2 isoform, which leads to stronger expression of key glycolytic proteins such as Glut1 and Mct4. In contrast to cortical astrocytes, hypothalamic astrocytes do not respond to glutamate with enhanced glycolytic flux. However, their lactate production is highly sensitive to glucose levels, a characteristic that depends on the expression of the intracellular energy sensor AMPK (AMP‐activated protein kinase). These features clearly establish the glucose‐sensing nature of hypothalamic astrocytes and explain the mechanism by which they can fulfill this role.

## 2 Materials and Methods

### 2.1 Animals

Animals were maintained in a temperature‐controlled animal facility with a 12‐h light/12‐h dark cycle and had access to food and water according to the Swiss Animal Protection Ordinance (OPAn). All experiments were approved by the Service Vétérinaire du Canton de Vaud, Switzerland (License No. 1251.5 and VD3733).

MCT4^flox/flox^ and MCT1^flox/flox^ mice were generated in collaboration with Cyagen Biosciences. The targeting vector included a Neo resistance cassette flanked by FRT sites, as well as Cre‐dependent lox P sites in introns 2 and 5 for MCT4^flox/flox^ mice and in introns 4 and 5 for MCT1^flox/flox^. C57BL/6 embryonic stem cells were used for gene targeting, and the positive cells were bred into albino B6 female mice. This strategy allowed us to have a pure B6 background. MCT4^flox/+^ mice and MCT1^flox/+^ mice were then crossed with mice expressing Flp recombinase (B6.Cg‐Tg[Pgk1‐FLPo]10Sykr/J) in order to remove the Neo resistance cassette. With one subsequent cross with B6 animals, the Flp transgene was removed, and the obtained MCT4^flox/+^ mice or MCT1^flox/+^ mice were then intercrossed in order to generate the MCT4^flox/flox^ and MCT1^flox/flox^ mice used in this study. These transgenic mouse lines have been previously used successfully to generate conditional KO animals after cell‐specific Cre recombinase expression (Braga et al. 2024; Martini et al. 2021; Netzahualcoyotzi and Pellerin 2020).

hGFAP‐eCFP (TgN(hGFAP‐ECFP)‐GCED) mice have been obtained from Dr. Frank Kirchhoff (Molecular Physiology, University of Saarland, Germany). In this model, astrocytes express enhanced cyan fluorescent protein (eCFP) under the human glial fibrillary acidic protein (hGFAP) promoter (Hirrlinger et al. 2005).

### 2.2 Primary Cultures of Mouse Hypothalamic and Cortical Astrocytes

Unless otherwise stated, all chemicals were purchased from Invitrogen (Luzern, Switzerland). Primary cell cultures were prepared from OF1: SWISS mice (Janvier Laboratories, France) or from MCT4^flox/flox^ mice and MCT1^flox/flox^ mice according to SCAV authorization (No. 1251.5). Astrocytes from the hypothalamus and cerebral cortex were obtained from the same animals and isolated, cultured, and treated at the same time with the same medium. Astrocytes were isolated from the cerebral cortex or hypothalamus of 2‐3 days old pups and cultured as previously described by Prevot et al. (2003). After decapitation, the brain was removed from the skull, and the meninges were excised. The cortex and hypothalamus were isolated, and cells were mechanically dissociated using 3 types of needles (1.1 × 40, 0.8 × 40, and 0.5 × 16 mm). Astrocytes were grown for 18–21 days in 75‐cm^2^ flasks containing DMEM/F12 medium supplemented with 10% fetal calf serum, 1% L‐glutamine, and 1% penicillin/streptomycin (P/S). Both cell types were then seeded in plates (composed of 6, 12, or 24 wells) or 35‐mm dishes, depending on the experiments, at a density of 55,000 cells/mm^2^. After 24 h, cells were incubated for 2 days in astrocyte‐defined medium composed of DMEM (5 mM glucose, without phenol red) supplemented with 1% L‐glutamine, 5 μg/mL insulin (Sigma, Buchs, Switzerland), and 100 μM Putrescine (Sigma, Buchs, Switzerland). Cultured cells were maintained in a water‐saturated atmosphere at 37°C with 5% O_2_:95% CO_2_.

### 2.3 Primary Cultures of Human Hypothalamic Astrocytes

The studies on human fetal tissue were approved by the French Agency for Biomedical Research (Agence de la Biomédecine, Saint‐Denis la Plaine, France, Protocol No.: PFS16‐002). Nonpathological human fetuses were obtained at 9–12 gestational weeks from voluntarily terminated pregnancies after written informed consent from the donors (Gynecology Department, Jeanne de Flandre Hospital, Lille, France). Primary cultures of human hypothalamic astrocytes were prepared as previously described (Sharif et al. 2009; Sharif and Prevot 2012). Each culture was derived from a single embryo. Briefly, after removal of the brain, the hypothalamus was dissected and mechanically dissociated into a single‐cell suspension by passage through a 20‐μm nylon mesh (Buisine). Dissociated cells were transferred to a 75‐cm^2^ culture flask and cultured in Dulbecco's Modified Eagle's Medium (DMEM)/F‐12 supplemented with 10% (v/v) fetal calf serum, 2 mM L‐glutamine, 100 units/mL penicillin, and 100 μg/mL streptomycin (all from Gibco) under a humid atmosphere of 5% CO_2_–95% air at 37°C. Culture medium was changed 1 week after initiating the culture and then once a week until reaching confluence. Astrocytes were grown, seeded in plates, and treated as described above for primary cultures of mouse astrocytes.

### 2.4 FACS Sorting

Cerebral cortex and hypothalamus were dissected from four P50‐old (postnatal day) males hGFAP‐eCFP mice, and samples were pooled and prepared as previously described (Cahoy et al. 2008; Zehnder et al. 2021). eCFP‐positive astrocytes were purified by fluorescence‐activated cell sorting (FACS) using a MoFlo AstriosEQ High‐speed cell sorter. hGFAP‐eCFP‐positive and eCFP‐negative sorted cells were lysed and homogenized in 350 μL of RP1 lysis Buffer (PrepEase RNA/Protein Spin Kit, Affymetrix, Staufen, Germany) in 3–4 replicates (*n* = 3–4) and conserved at −80°C until RNA extraction.

### 2.5 Cell Treatments

Astrocytes obtained from MCT4^flox/flox^ and MCT1^flox/flox^ mice were treated with 4 μM of TAT‐CRE recombinase protein (Millipore, Zug, Switzerland) or TAT‐FITC recombinase protein (Heulot et al. 2016) for 6 h, before being incubated in astrocyte‐defined medium for 2 days.

Glutamate (200 μM, Sigma, Buchs, Switzerland) treatments were performed in astrocyte‐defined medium. 5‐Aminoimidazole‐4‐carboxamide ribonucleoside (AICAR, 500 μM, 1 h, Selleck Chemicals, Euromedex, Souffelweyersheim, France) and Compound C (10 μM, 12 h, MedChem Express, Lucerna‐Chem AG Switzerland, Luzern, Switzerland) treatments were performed in DMEM (devoid of glucose and phenol red) supplemented with 1 mM up to 10 mM of glucose (Sigma, Buchs, Switzerland) depending on the experiments.

### 2.6 siRNA Transfection

After 18–21 days in 75‐cm^2^ flasks, astrocytes were transfected in suspension with small interfering RNA (siRNA) against PKM2 (siPKM2, 50 nM, Qiagen, Hombrechtikon, Switzerland) or a negative control (siCtr, 50 nM, Qiagen, Hombrechtikon, Switzerland) using Lipofectamine 3000 reagent (Invitrogen, ThermoFisher Scientific, Basel, Switzerland) prepared in Opti‐MEM/GlutaMax (Invitrogen, Basel, Switzerland) and DMEM F/12 medium without antibiotics according to the manufacturer's instructions. After 12 h of incubation, the transfection medium was replaced with DMEM F/12 medium. Astrocytes were harvested in astrocyte‐defined medium between 24 and 96 h (precise times are indicated in figure legends) before being collected for further analysis.

### 2.7 Glucose Uptake

^3^H‐2‐DG uptake was determined as previously described with minor modifications (Pellerin and Magistretti 1994). After 48 h of incubation in astrocyte‐defined medium, the medium was replaced by 2 mL of astrocyte‐defined medium containing ^3^H‐2‐DG (ARC, St Louis, US) at a concentration of 1 μCi/mL (specific activity, 60 Ci/mM). ^3^H‐2‐DG uptake results were calculated by subtracting from total counts the portion that was not inhibited by the glucose transporter inhibitor cytochalasin B (25 μM, Sigma, Buchs, Switzerland). Protein content was measured with a Pierce bicinchoninic acid assay (ThermoFisher Scientific, Basel, Switzerland), and glucose uptake values were expressed as fmol per mg of protein.

### 2.8 Lactate Release Assay

For the lactate release assay, the astrocyte‐defined medium was replaced with fresh astrocyte‐defined medium or, for the glucose concentration variation study, with DMEM (without phenol red and glucose) supplemented with appropriate concentrations of glucose. Then, cells were maintained in a water‐saturated atmosphere at 37°C with 5% O_2_:95% CO_2_. After the appropriate time of incubation, the medium was harvested for lactate measurement while the protein content was determined using a Pierce bicinchoninic acid assay (ThermoFisher Scientific, Basel, Switzerland). Lactate release into the medium was measured enzymatically using the L‐Lactate Assay Kit (Abcam ab65330) or, for the glucose concentration variations study, by the method of Rosenberg and Rush (1966). Both methods exhibit the same sensitivity. Briefly, the culture medium was mixed with a 0.33 M glycine–semicarbazide supplemented buffer, just before starting the 1 h incubation period at 37°C, with 0.015 M nicotinamide adenine dinucleotide (Roche, Basel, Switzerland) and 70 U/mL l‐lactate dehydrogenase (Roche, Basel, Switzerland). The quantification was performed by measuring the NADH produced via the L‐lactate dehydrogenase‐catalyzed enzymatic reaction, with a microplate reader (SynergyMx; BioTek, Luzern, Switzerland) at 340 nm. For the L‐lactate Assay Kit, fluorescence (Ex/Em = 535/590 nm) emitted by the NADH interaction with the lactate probe was also quantified using a microplate reader (SynergyMx; BioTek, Luzern, Switzerland). Released lactate values were normalized to the total protein content for each well. Finally, lactate release was expressed in μmol/mg protein.

### 2.9 Seahorse Analyses

For Seahorse analyses, cells were seeded 3 days before the experiment in XF24‐well cell culture microplates coated with poly‐D‐lysine (5 µg/ml, Sigma, Buchs, Switzerland) at a density of 6 × 10^4^ cells per well in 200 μL DMEM/F12 media. After 24 h, the medium was replaced with astrocyte‐defined medium. Cells were incubated for an additional 2 days at 37°C and 5% CO_2_ prior to the assay. Extracellular acidification rate (ECAR) and oxygen consumption rate (OCR) were measured in adherent astrocytes with an XF‐24 extracellular flux analyzer (Seahorse Bioscience, Agilent, Santa Clara, United States). Just before the experiment, cells were washed, and the medium was replaced with DMEM (without phenol red and glucose) containing 2 mM glutamine and appropriate glucose concentrations (0.1 or 1 mM). Cells were then preincubated for 1 h at 37°C without CO_2_ to allow cells to pre‐equilibrate with the assay medium before starting the procedure. After measuring baseline ECAR and OCR, both were assessed following an acute injection of the appropriate substrate(s) or drug(s). ECAR was expressed as mpH per minute; OCR was expressed as pmol of O_2_ per minute. Both were normalized by protein content, which was measured with a Pierce bicinchoninic acid assay (ThermoFisher Scientific, Basel, Switzerland). The number of wells used (*n*) per experiment (*N*) is specified in the figure legends.

### 2.10 Calcium Imaging

Hypothalamic astrocytes were plated 3 days before the experiments in 35‐mm glass bottom MatTek dishes with a 12‐mm‐diameter coverslip adapted for microscopic acquisition (Nunc, Thermo Fisher Scientific, Basel, Switzerland) coated with poly‐D‐lysine (5µg/ml, Sigma, Buchs, Switzerland) and incubated at 37°C in 5% CO_2_ in a humidified atmosphere. Twenty‐four hours later, the medium was replaced by astrocyte‐defined medium, and cells were incubated for two more days. Cells were then washed (with PBS) and incubated in 5 mM glucose phenol red free DMEM medium (5 mM, Sigma, Buchs, Switzerland) containing the cytosolic Ca^2+^ indicator Fluo‐4/AM (5 μM, Invitrogen, ThermoFisher Scientific, Basel, Switzerland) for 20 min in the incubator. Just before Ca^2+^ fluxes recording, the Fluo‐4/AM medium was removed, and the dishes were washed and incubated with DMEM medium containing glucose (5 mM). Fluorescence was monitored using a time‐lapse imaging mode on a confocal microscope system (Zeiss, Feldbach, Switzerland; X40 oil immersion lens; the excitation wavelength was 488 nm, and the emitted fluorescence was recorded between 495 and 525 nm). After recording basal fluorescence in 5 mM glucose, cells were stimulated with DMEM medium containing different glucose concentrations (5 or 10 mM). Data were analyzed using the Zen software (Zeiss, Feldbach, Switzerland). The use of the single excitation/emission Fluo‐4 dye necessitated normalizing each time point fluorescence (F) to time zero fluorescence (F0) in each cell to account for possible differences in dye loading and excitation strength as previously reported (Nat Comm Zanou et al. 2021, Vidal et al. 2023). The basal fluorescence was subtracted from the maximal fluorescence in response to the stimulation to obtain *ΔF* corresponding to the amplitude of the response. *ΔF*/F0 values represented in the results correspond to the mean of ΔF/F0 for all cells (*n*) from 3–4 wells per condition (N).

### 2.11 Immunocytochemistry

Immunofluorescence labelings were performed essentially as previously described by Geller et al. (2019). Astrocytes incubated for 48 h in astrocyte‐defined medium were fixed for 20 min in 4% paraformaldehyde at room temperature (RT). Fixed cells were incubated in blocking buffer, composed of PBS 0.3% Triton X‐100 (Sigma, Buchs, Switzerland) and 2% donkey serum (Sigma, Buchs, Switzerland), for 30 min at RT. Immunostainings were carried out in blocking buffer with the different antibodies (i) overnight at 4°C with rabbit anti‐MCT4 (1:250; #sc‐50,329; SantaCruz, Heidelberg, Germany), mouse anti‐S100β (1:500; #AMAB91038, Sigma‐Aldrich, Buchs, Switzerland), or rabbit anti‐glial fibrillary acidic protein (GFAP) (1:500; #Z0334, DakoCytomation, Baar, Switzerland); (ii) three nights at 4°C with rabbit anti‐PKM2 (1:20; #15822–1‐AP, Proteintech, Manchester, United Kingdom). Then, fixed cells were incubated in blocking buffer containing a donkey anti‐rabbit AF594 antibody (1:500; Jackson Immunoresearch, Suffolk, UK) and a donkey anti‐mouse antibody AF488 (1:500; Jackson Immunoresearch, Suffolk, UK) or an Alexa Fluor 488 antiphalloidin (1:100; #A12379, Invitrogen, ThermoFisher Scientific, Basel, Switzerland) for 2 h at RT. Cells were incubated for 2 min with nuclear counterstain Hoechst 33258 (1/10,000; #H3569, Thermo Fisher Scientific, Basel, Switzerland) before being mounted onto glass slides in Fluoromount (Sigma, Buchs, Switzerland). Fluorescence images were captured using an inverted Zeiss LSM 710 Quasar Confocal Microscope (Zeiss, Feldbach, Switzerland).

### 2.12 RNA and Protein Purification

Proteins were extracted with RIPA lysis buffer (Millipore, Zug, Switzerland), completed with protease/phosphatase inhibitors (Thermo Scientific, Reinach, Switzerland). Astrocyte cultures and sorted cells were lysed and homogenized in 350 μL of RP1 lysis buffer (PrepEase RNA/Protein Spin Kit, Affymetrix, Staufen, Germany). Total RNA from both primary cultures and sorted cells was purified using the RNeasy Mini Kit (Qiagen, Basel, Switzerland) according to the manufacturer's instructions. RNA was digested with DNase (RNase‐Free DNase Set, Qiagen, Basel, Switzerland) and eluted with 20 μL of water. RNA concentrations were determined with a Nanodrop (ND‐1000 Spectrophotometer, Witec, Luzern, Switzerland). Protein concentrations were determined using the Micro BCA Protein assay kit (Invitrogen, ThermoFisher Scientific, Basel, Switzerland), according to the manufacturer's instructions.

### 2.13 RT‐qPCR

RNA reverse transcription and real‐time PCR were performed as described by Geller et al. (2019). Briefly, RNA was reverse transcribed using the RT high‐capacity RNA‐to‐cDNA kit (Applied Biosystems, Rotkreuz, Switzerland) at final concentrations of 4 and 3.6 ng/μl for primary cultures and sorted cells, respectively, and real‐time PCR analysis was conducted on 2 μL of the cDNA with Power SYBR Green Taq polymerase master mix (Applied Biosystems, Rotkreuz, Switzerland). Primer sequences for mRNA quantification and the housekeeping gene Poly2a (polymerase 2a) are listed in Table 1.

**TABLE 1:** Primer sequences used to perform RT‐qPCR of various target genes of key metabolic astrocyte proteins.

Gene symbol (protein name)	Primer sequences (5′‐3′)
*Slc2a1* (Glut1)	GCCCCCAGAAGGTTATTG
	CGTGGTGAGTGTGGTGGATG
*Slc2a2* (Glut2)	TTCCGGAAGAAGAGTGGTTCG
	TGGTCGGTTCCTCGG TTTTAG
*Hk1* (Hk1)	CATTGTCTCCTGCATCTCCGA
	ATTCGGCAATCTAGGCTCGTC
*Hk2* (Hk2)	CTTGCGAATATGGTTGCCTCA
	CCTCCAATGCCTGATCATCTGAT
*Gck* (Hk4, Gk)	CATCCTGCTCAACTGGACCAA
	CATTGCCACCACATCCATCTC
*Pkm1* (Pkm1)	ACT CCG TGA GAA CTA TCA AAG C
	TTG TGC GAG CCT CCA GTC
*Pkm2* (Pkm2)	TTGCAGCTATTCGAGGAACTCCG
	CACGATAATGGCCCCACTGC
*Ldhb* (Ldh1)	ATTGCGTCCGTTGCAGATG
	TCCCAGAATGCTGATGGCA
*Ldha* (Ldh2)	CAAAGTCCAAGATGGCAACCC
	AGCACCAACCCCAACAACTGT
*Slc16a1* (Mct1)	TTGGACCCCAGAGGTTCTCC
	AGGCGGCCTAAAAGTGGTG
*Slc16a3* (Mct4)	GTGTCGCTGTAGCCAATCCC
	GGCTGTTTTATCATCACGGGT
*eCfp* (CFP)	TACCCCGACCAGATGAAGCA
	CCGTCGTCCTTGAAGAAGATGGT
*Cre*	TTGGCAGAACGAAAACGCTG
	TTGGCAGAACGAAAACGCTG
*Gfap* (GFAP)	ACGACTATCGCCGCCAAC
	GCGGCTCTAGGGACTCGTTC
*Atp1a2* (Na^+^/K^+^ ATPase α2)	GAGACGCGCAATATCTGTTTCTT
	ACCTGTGGCAATCACAATGT
*Polr2a* (Poly2a)	CCCTCATCATACCTGGACACATC
	GTAAGGGCCACTATCTTCATCATCA

### 2.14 Western Blotting

Western blots were performed essentially as described by Geller et al. 2019. Proteins were separated with 7.5%, 10%, or 15% homemade SDS‐PAGE or precast gels (4%–15% or 8%–16% Bio‐Rad, Reinach, Switzerland). After transfer to nitrocellulose or PVDF membranes, detection of protein transfer efficiency was performed using reversible protein stain (see below for normalization). Membranes were incubated in blocking buffer composed of TBST 5% nonfat milk (Sigma, Buchs, Switzerland) or 5% BSA (Sigma, Buchs, Switzerland) for 30 min at RT. Then, membranes were incubated with primary antibody (see Table 2 for references and dilutions) overnight at 4°C and for 2 h at RT with goat anti‐rabbit or goat anti‐mouse peroxidase‐conjugated secondary antibody (1/5000; GE Healthcare, Piscataway, NJ, USA) or rabbit anti‐goat peroxidase‐conjugated secondary antibody (1/5000; Jackson Immunoresearch, Suffolk, UK).

**TABLE 2:** Antibodies used to perform western blot of various target proteins of key metabolic astrocyte proteins.

Antibody	Host	Dilution	Source
AMPK‐α	Rabbit	1:1000	Cell Signaling 2532S
P‐AMPK‐α T172 (40H9)	Rabbit	1:1000	Cell Signaling 2535S
ACC (Acetyl‐CoA carboxylase)	Rabbit	1:1000	Cell Signaling 3662S
P‐ACC Ser79	Rabbit	1:1000	Cell Signaling 3661S
ErBb1 (Egfr)	Goat	1:250	Santa Cruz sc‐036
ErBb4	Rabbit	1:1000	Santa Cruz sc.283
GK (Gck)	Sheep	1:1000	Denechaud et al. 2008
Glast	Rabbit	1:5000	Shibata et al. 1997
Glt1	Rat	1:250	Alphadiagnostic GLT11‐A
Glut1	Rabbit	1:2000	Millipore 07–1401
Glut2 (H‐67)	Rabbit	1:1000	Santa Cruz sc‐9117
Glutamine synthetase (GS)	Rabbit	1:1000	Abcam ab73593
HK1	Rabbit	1:1000	Millipore AB3543
HK2	Rabbit	1:1000	Cell signaling 2867 C64G5
Lamini A‐C (H110)	Rabbit	1:1000	Santa Cruz sc‐20,681
Ldh1	Rabbit	1:1000	Millipore AV48210
Ldh5	Rabbit	1:1000	Cell Signaling 2012S
Mct1	Rabbit	1:1000	Pierre et al. 2000
Mct4 (H‐90)	Rabbit	1:250	Santa Cruz sc‐50,329
NaKATPase α2	Rabbit	1:250	Cholet et al. 2002
NeuN	Rabbit	1:500	Millipore ABN78
Pkm1	Rabbit	1:2000	Novus Biologicals NBP2‐14833
Pkm2	Rabbit	1:1000	Proteintech 15,821–1‐AP
von Willebrand factor	Rabbit	1:1000	DAKO A082

Bands were revealed with a chemiluminescence kit, ECL or Sirius (BioRad, Reinach, Switzerland) and processed with a ChemiDoc XRS system (BioRad, Reinach, Switzerland) for densitometry analysis. For phosphorylated AMPK and ACC immunoassays, membranes were blocked using the LI‐COR blocking buffer (LI‐COR, Lincoln, NE, USA) before primary antibody incubation. Membranes were then washed and incubated with IRDye 800‐conjugated donkey anti‐rabbit IgG (LI‐COR, Lincoln, NE, USA, 1:5000) or IRDye 680‐conjugated donkey anti‐mouse IgG (LI‐COR, Lincoln, NE, USA, 1:10,000). Immunoreactive bands were visualized using infrared fluorescence (IR‐Odyssey scanner, LI‐COR, Lincoln, NE, USA). Band densities were quantified using Image Studio v 5.2.5 (LI‐COR, Lincoln, NE, USA).

When two proteins of interest were to be detected on the same membrane, the membrane was stripped after detection of the first target protein. For membranes developed using chemiluminescence, stripping was performed by incubating the membrane in a stripping buffer composed of 6% 1 M HCl (pH 6.8), 0,8% β‐mercaptoethanol, and 4% SDS for 30–45 min at 50°C with constant agitation. For membranes revealed using fluorescence, the commercial LI‐COR NewBlot IR Stripping Buffer (LI‐COR Biosciences) was used according to the manufacturer's protocol.

Western blots were normalized to total protein levels using the MemCode Reversible Protein Stain Kit (ThermoFisher Scientific) or LiCor total revert protein stain (LI‐COR Biosciences) or to β‐actin, as indicated in the figures. For illustration purposes, black and white images were uniformly adjusted for contrast and brightness across the entire image. Cropped regions are indicated by bold vertical lines.

### 2.15 HR‐MAS NMR Spectroscopy

Male Wistar RJ‐HAN (200 g, Janvier Laboratories, France) were used for NMR experiments. Rats were rapidly euthanized by brain‐focused microwaves (5 KW, 1 s, Sacron 8000, Sairem), the only method capable of immediately halting all enzymatic activity and preventing post‐mortem artifacts, such as anaerobic lactate production, as demonstrated by Roumes et al. (2021). Two biopsies, each approximately 20 mg, were collected from each rat: one from the S1BF area (barrel cortex) and one from the medio‐basal hypothalamic region. To ensure precise and reproducible dissection of the targeted brain regions, a rat brain matrix was used. The biopsies were immediately immersed in liquid nitrogen and stored at −80°C until NMR analysis. HR‐MAS enables the acquisition of spectra with high spectral resolution directly from the biopsies. The biopsies were weighed and placed directly in the HR‐MAS rotor along with 30 μL of D_2_O containing a known amount of ethylene glycol, used as an external reference. ^1^H‐NMR spectra were acquired with a 90° pulse angle (adjusted for each sample), a relaxation time of 8 s, a spectrum width of 10 ppm (ppm), an acquisition time of 3.28 s, 128 scans, and a memory size of 32 K. The water signal was suppressed using homonuclear presaturation. Spectra were analyzed and quantified using TopSpin 3.2.

### 2.16 Statistical Analysis

All results are presented as mean ± SEM. For ex vivo studies, the “N” indicated in figure legends corresponds to the number of animals per group. For in vitro studies, experiments were performed on three independent cultures (*N* = 3) and at least three wells per culture (*n* = 3), except as stated otherwise in figure legends. Data were analyzed with GraphPad Prism 10.02.2 (San Diego, CA, USA). Statistical details are indicated in figure legends. Outliers were estimated using GraphPad QuickCals outlier calculator with a significance level of *p* < 0.05 (https://www.graphpad.com/quickcalcs/Grubbs2). After verifying the normal distribution of the data, one‐way ANOVA followed by Dunnett's multiple comparisons test against the control, or two‐way ANOVA followed by Tukey's or Sidak's test for multiple comparisons, was performed where appropriate. For simple comparison, when a normal distribution was observed, an unpaired or paired *t*‐test was performed. Otherwise, a Mann–Whitney test or a Wilcoxon test (to compare two paired samples) was applied. *p* ≤ 0.05 was considered statistically significant.

## 3 Results

**FIGURE 1:**
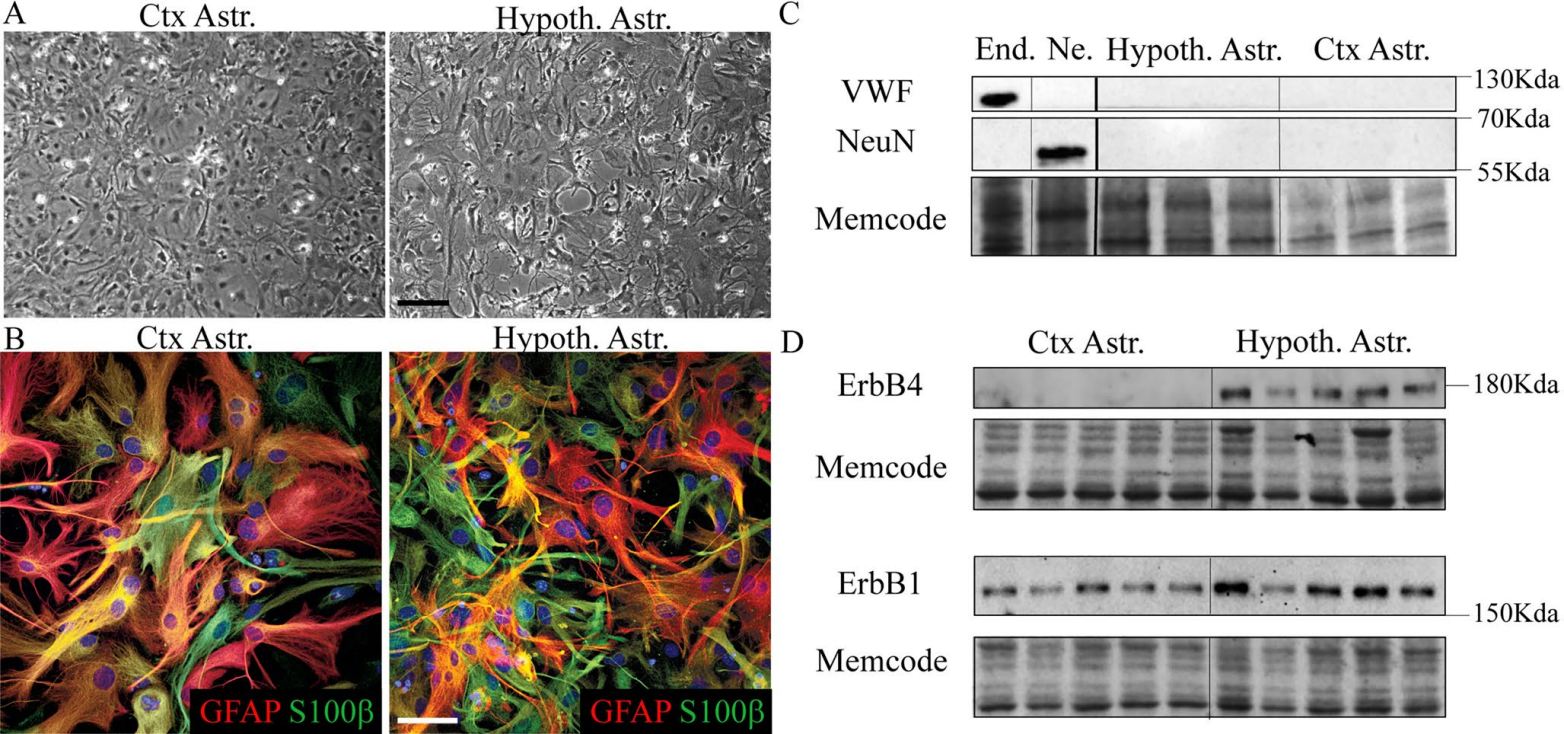
Phenotypic characterization of primary cultures of mouse cortical and hypothalamic astrocytes. A. Differential interference contrast (DIC) photomicrographs showing astrocytes from cerebral cortex (Ctx Astr.) and hypothalamus (Hypoth. Astr.) B. Immunofluorescence photomicrographs showing immunoreactivity for the astrocytic markers GFAP (red) and S100β (green) in cultured cortical and hypothalamic astrocytes C. Representative western blots for the endothelial marker von Willebrand factor (VWF) and the neuronal marker NeuN in mouse endothelial cells (End.), mouse neurons (Ne.) as well as in hypothalamic and cortical astrocytes D. Representative western blots of ErbB expression profiles in cortical and hypothalamic astrocytes. Scale bars (A) 200 μm and (B) 50 μm.

To better characterize the metabolic phenotype of hypothalamic astrocytes and compare it to cortical astrocytes (Ctx Astr.), primary cultures of cortical and hypothalamic astrocytes were prepared from mouse pups and investigated first for the expression of various markers. Differential interference contrast (DIC) microphotographs (Figure 1A) illustrate the distinct morphology of mouse cortical (Ctx Astr.) and hypothalamic (Hypoth. Astr.) astrocytes after 3 weeks in culture, as also previously described in astrocyte cultures from humans (Sharif et al. 2009; Sharif and Prevot 2012). Cells from both areas were immunolabeled for both Gfap and S100β, two astrocytic markers (Figure 1B). Then, the purity of these primary cultures of astrocytes was determined (Figure 1C). Western blot analysis demonstrated the absence of the neuronal marker NeuN and the endothelial marker Von Willebrand Factor (VWF) in both astrocyte cultures. Both primary cultures of mouse cortical and hypothalamic astrocytes expressed the ErbB1 receptor (Figure 1D). In contrast, ErbB4 was detected only in hypothalamic astrocytes and not in cortical astrocytes (Figure 1D), as previously described in vitro and in vivo (Sharif and Prevot 2010). These results not only confirm that each region‐specific primary culture is enriched in astrocytes but also show the capacity of hypothalamic and cortical astrocytes to maintain in vitro the differential molecular phenotype that they exhibit in vivo.

### 3.1 Hypothalamic Astrocytes Exhibit a Stronger Glycolytic Phenotype Compared to Cortical Astrocytes

**FIGURE 2:**
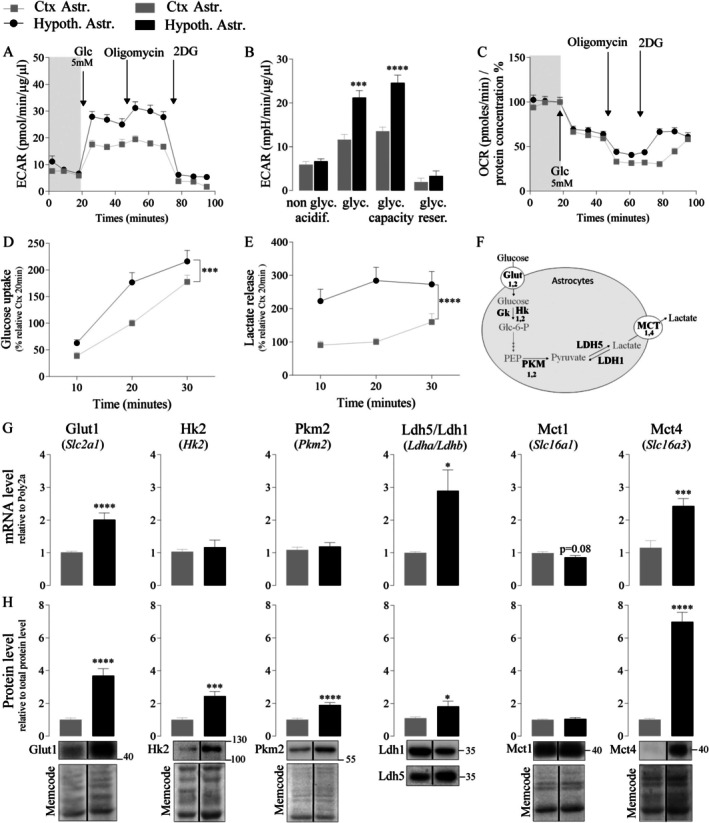
Characterization of the glycolytic profile of cortical and hypothalamic astrocytes. A‐C. ECAR (A‐B) and OCR (C) levels of cortical and hypothalamic astrocytes before (Gray frames, 0.1 mM glucose) and after adding 5 mM glucose, Oligomycin (1 μM) and 2DG (50 mM) (*n* = 10) D. Time‐dependent ^3^H‐2‐Deoxyglucose (2DG) uptake by cortical and hypothalamic astrocytes (*N* = 3, *n* = 3–5) E. Time‐dependent lactate accumulation in the culture medium of cortical and hypothalamic astrocytes (*N* = 3, *n* = 3–5) F. Schematic representation of glycolysis with the steps regulated by the investigated elements in astrocytes. G‐H. mRNA expression levels (G) and western blot analysis (H) of protein expression levels in cortical and hypothalamic astrocytes (*N* = 3–6, *n* = 3–5). Cropped membrane images of Western blots originate from original figures in Data S1. Statistical analysis was performed using two‐way ANOVA followed by Sidak's multiple comparisons test. For simple comparison, unpaired *t*‐test or Mann–Whitney test was performed. *p < 0.05; **p < 0.01; ***p < 0.001; ****p < 0.0001. Values indicate means ± SEM. See also Figure S1.

We first explored the metabolism of astrocytes from both areas using the Seahorse flux analyzer, with the sequential addition of glucose (from 0.1 to 5 mM), oligomycin, and 2‐Deoxy‐D‐glucose (2DG) (Figures 2A,B,C and S1A,B). Astrocytes from cortex and hypothalamus had a similar extracellular acidification rate (ECAR) at 0.1 mM glucose (gray zone, Figure 2A; considered as nonglycolytic acidification, unpaired *t*‐test *p* = 0.40, Figure 2B). After adding 5 mM of glucose, both cell types exhibited an increase in ECAR, confirming that astrocytes from both areas have a substantial glycolytic profile (Figure 2A). However, hypothalamic astrocytes presented an ECAR enhancement about two‐fold larger compared to cortical astrocytes (considered as essentially due to glycolysis, unpaired *t*‐test, *p* < 0.0001, Figure 2B). To determine the maximal glycolytic capacity of both cell types, ATP synthase was inhibited by adding oligomycin, a complex V inhibitor. It is interesting to note that, in parallel, the oxygen consumption rate (OCR) was reduced in both cell types upon oligomycin addition, showing the efficiency of the treatment (Figure 2C). Only hypothalamic astrocytes presented a small increase in ECAR after adding oligomycin (Figure 2A), and thus glycolytic capacity remained larger compared to cortical astrocytes (unpaired *t*‐test, *p* < 0.0001, Figure 2B). No significant difference in glycolysis reserve (which corresponds to the difference between glycolytic capacity and glycolysis, unpaired *t*‐test, *p* = 0.3491, Figure 2B) was detected. Finally, after adding 2DG (an inhibitor of glycolysis), astrocytes from both areas presented a strong decrease in ECAR with the same residual level (Figure 2A), confirming that the ECAR modifications observed with the Seahorse flux analyzer protocol are dependent on glucose utilization by these cells. Measurement of the OCR revealed another interesting feature. The addition of 5 mM glucose significantly reduced OCR to the same magnitude in both cell types (Figure 2C, S1B). This decrease in oxygen consumption after increasing the concentration of glucose from 0.1 to 5 mM favors glycolysis and thus reveals a genuine Crabtree effect in astrocytes from both regions (Barros et al. 2021), emphasizing their glycolytic preference. To confirm these initial observations, glucose utilization and lactate release by hypothalamic astrocytes were studied and compared to cortical astrocytes at 5 mM of glucose (Figure 2D,E). ^3^H‐2DG uptake showed a higher glucose utilization by hypothalamic astrocytes versus cortical astrocytes over time (two‐way ANOVA; cell type *p* = 0.0001, time effect *p* < 0.0001, no interaction; Figure 2D). In parallel, the amount of lactate released in the culture medium by hypothalamic astrocytes was at least twice as much over time compared to cortical astrocytes (two‐way ANOVA; cell type *p* < 0.0001, no time effect or interaction, Figure 2E).

The levels of mRNA and protein expression for the key transporters and main enzymes involved in glycolysis (Figure 2F) were compared between astrocytes from both areas using qPCR and western blot analyses (Figures 2G,H and S1). Hypothalamic astrocytes presented higher mRNA and protein expression levels for the glucose transporter 1 (unpaired *t*‐test, *Slc2a1 p* < 0.0001, Glut1 p < 0.0001, Figures 2G,H and S1C–H). No difference was observed between cortical and hypothalamic astrocytes for *Slc2a2* mRNA expression and Glut2 protein production (unpaired *t*‐test, respectively *p* = 0.15 and *p* = 0.62, Figure S1D). Among the enzymes involved in glucose metabolism (hexokinase 1 and 2 [Hk1, Hk2] or glucokinase [Gk]), only the Hk isoform 2 protein was more highly expressed in hypothalamic astrocytes (Mann–Whitney test *Hk2 p* = 0.41, unpaired *t*‐test Hk2 *p* = 0.0004, Figures 2G,H and S1E, Mann–Whitney test *Hk1 p* = 0.08, unpaired *t*‐test Hk1 *p* = 0.29 Figure S1D, unpaired *t‐*test *Gk p* = 0.07, Mann–Whitney test Gk *p* = 0.11, Figure S1D,E). The isoform M2 but not M1 of the pyruvate kinase (PK) protein was more expressed in hypothalamic astrocytes (Mann–Whitney test, *Pkm2 p* = 0.95, unpaired *t*‐test Pkm2 *p* < 0.0001, *Pkm1* p = 0.15, Pkm1 *p* = 0.449, Figures 2G,H and S1D,F). The mRNA expression levels of lactate dehydrogenase subunits were also determined: *Ldhb*, giving rise to the subunit present in the Ldh1 isoform (enzyme favoring pyruvate production, Figure 2F), and *Ldha*, giving rise to the subunit present in the Ldh5 isoform (enzyme favoring lactate production, Figure 2F). The ratios of *Ldha/Ldhb* mRNAs and Ldh5/Ldh1 proteins were determined in order to obtain a first hint of the capacity of cells to produce lactate from pyruvate (Figure 2G,H). Both ratios were higher in hypothalamic astrocytes (Mann–Whitney test *Ldha/Ldhb p* = 0.013, unpaired *t‐*test Ldh5/Ldh1 *p* = 0.03), suggesting they have a greater capacity to produce lactate than cortical astrocytes. Finally, hypothalamic and cortical astrocytes did not display a significant difference in either mRNA or protein expression levels for Mct1 (unpaired *t‐*test *Slc16a1 p* = 0.08, Mct1 p = 0.6, Figures 2G,H and S1G). In contrast, hypothalamic astrocytes expressed higher mRNA and protein levels of the transporter Mct4 (unpaired *t*‐test *Slc16a3 p* = 0.0003, Mct4 *p* < 0.0001, Figures 2G,H and S1H).

Altogether, these data suggest that astrocytes from distinct brain areas exhibit a prominent glycolytic capacity. However, hypothalamic astrocytes present functional and molecular characteristics consistent with a stronger glycolytic profile than cortical astrocytes.

### 3.2 Mct4 is the Main Lactate Transporter of Hypothalamic Astrocytes

Among the enzymes and transporters that most likely determine the strong glycolytic phenotype of hypothalamic astrocytes, Mct4 represents a key element. Indeed, its expression was often associated with tissues exhibiting a high glycolytic activity (e.g., white muscle fibers). Moreover, it is known that Mct4 is poorly detected in cortical astrocytes under standard culture conditions with a high oxygen environment (i.e., 21%), but its expression increases together with the glycolytic capacity at lower oxygen levels (Rosafio and Pellerin 2014).

**FIGURE 3:**
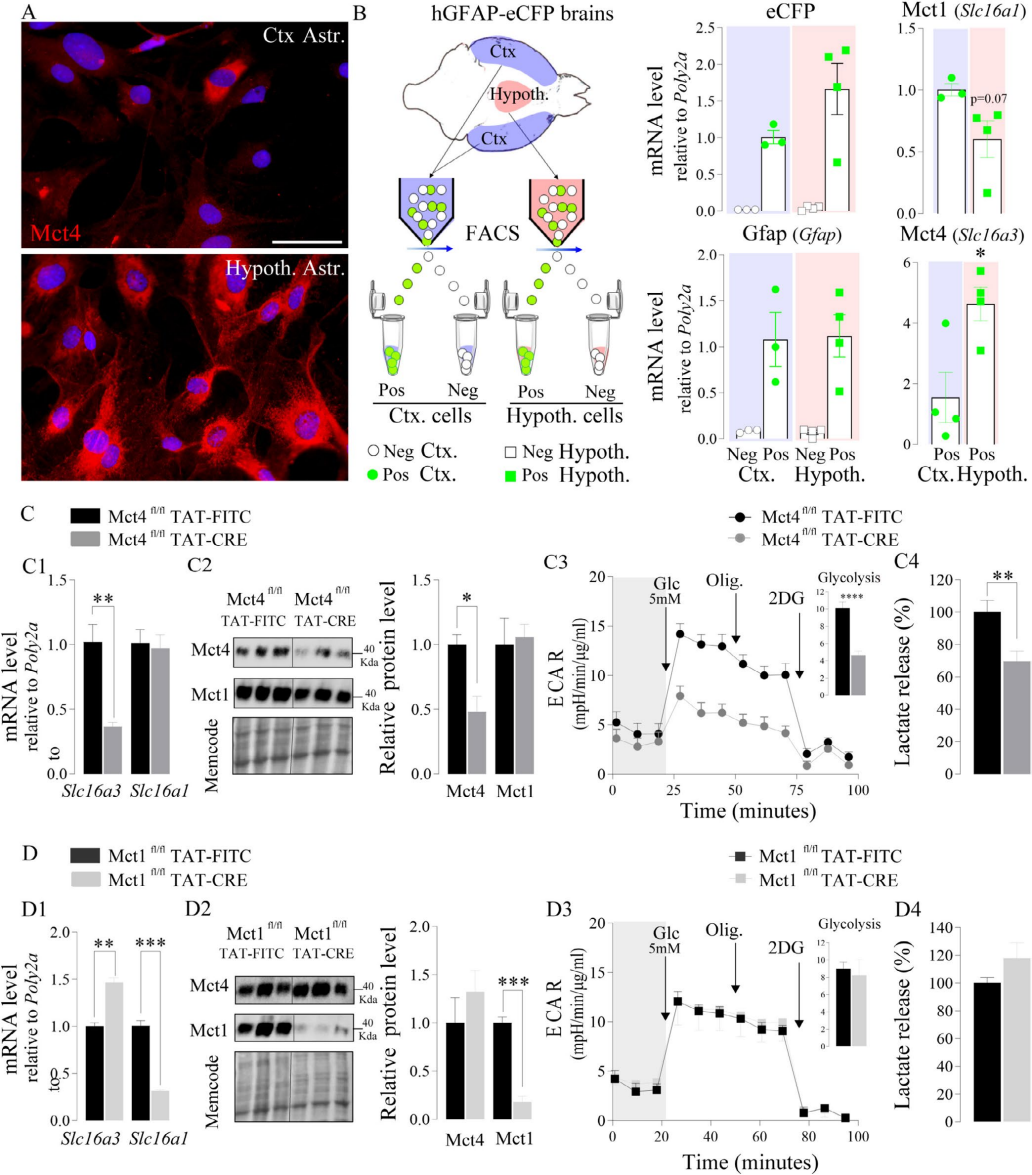
Mct1 and Mct4 expression in hypothalamic astrocytes and their importance for the prominent glycolytic profile of these cells. A. Immunofluorescence photomicrographs showing Mct4 immunoreactivity (red) in cultured cortical (top panel) and hypothalamic (bottom panel) astrocytes. Nuclei were stained with Hoechst (blue) B. Experimental procedure for isolation of cortical (violet) and hypothalamic (pink) astrocytes by FACS and RT‐qPCR analysis of e*Cfp*, *Gfap*, *Slc16a1*, *Slc16a3* expression in [eCFP^+^] cells (Pos, astrocytes) and/or [eCFP‐] cells (Neg) from both brain regions. C‐D. Analysis of Mct1/4 expression by RT‐qPCR (C1, D1) and by western blots (C2, D2); analysis of acidification rate (C3, D3, *n* = 5–11) and extracellular lactate concentration (C4, D4, *N* = 2, *n* = 3) of hypothalamic astrocyte cultures from Mct4^fl/fl^ mice (C) or Mct1^fl/fl^ mice (D) treated with Tat‐FITC (control, dark) or Tat‐Cre (pale). Statistical analysis was performed using unpaired *t*‐test. *p < 0.05; **p < 0.01; ***p < 0.001. Values indicate means ± SEM. Scale bars (A) 50 μm. See also Figure S2.

Western blot and immunofluorescence analysis confirmed that the Mct4 protein expression in hypothalamic astrocytes is more important than in cortical astrocytes in vitro under identical standard culture conditions (Figures 3A and S1H). To determine if the strong Mct4 protein expression in hypothalamic astrocyte cultures reflects an intrinsic characteristic of these cells in vivo, cortical and hypothalamic astrocytes from hGFAP‐eCFP mice were isolated by FACS (Figures 3B and S2A). qPCR performed on [GFAP‐eCFP^+^] cells (Pos) and [GFAP‐eCFP^−^] cells (Neg) detected strong *eCFP* and *GFAP* expression in Pos cells from both the cortex and hypothalamus, compared to Neg cells (Figure 3B), confirming the astrocytic enrichment of Pos cells. [GFAP‐eCFP^+^] cells from cortex and hypothalamus did not present a significant difference, but a tendency for lower expression levels of *Slc16a1* in hypothalamic astrocytes was noticed (unpaired *t‐*test, *p* = 0.07, Figure 3B), as described in vitro (unpaired *t‐*test, p = 0.08, Figure 2G). In contrast, *Slc16a3* mRNA expression was significantly higher in [GFAP‐eCFP^+^] cells from the hypothalamus compared to [GFAP‐eCFP^+^] cells from the cortex (unpaired *t‐*test, *p* = 0.02, Figure 3B), confirming the aforementioned in vitro results (Figure 2G).

The potential implication of Mct4 and Mct1 in the high capacity of hypothalamic astrocytes to release lactate was investigated in vitro. Previously developed Mct4^fl/fl^ and Mct1^fl/fl^ mice (Figure S2B,C) were used to prepare primary cultures of hypothalamic astrocytes. Deletion of either Mct4 or Mct1 in hypothalamic astrocyte cultures was obtained by treating cultured cells with a TAT‐CRE recombinase protein (Figure 3C,D). TAT‐FITC treatment was used as a negative control. Mct4^fl/fl^ astrocytes treated with TAT‐CRE exhibited a significant reduction of Mct4 mRNA and protein without any impact on Mct1 when compared to TAT‐FITC‐treated cells (unpaired *t‐*test, *Slc16a3 p* = 0.009, *Slc16a1 p* = 0.8, Mct4 *p* = 0.02, Mct1 *p* = 0,80 Figure 3C1,C2), thus demonstrating the specificity and efficiency of the deletion. Hypothalamic astrocytes with reduced expression of Mct4 (−64%) presented a significant decrease of glycolysis as revealed by ECAR measurement as well as lactate release (unpaired *t‐*test, Glycolysis *p* < 0.0001, lactate release *p* = 0.01, Figure 3C3,C4). In contrast, a comparable reduction in expression of Mct1 in hypothalamic astrocytes (−69%, unpaired *t‐*test, *Slc16a1 p* = 0.0002, Mct1 *p* = 0.0007, Figure 3D1,D2) did not have an impact on the glycolytic profile and lactate release (unpaired *t‐*test, glycolysis *p* = 0.72, lactate release, *p* = 0.162, Figure 3D3,D4). Interestingly, a significant increase in *Slc16a3* mRNA expression was observed in MCT1^fl/fl^ TAT‐CRE cultures (Mct4, unpaired *t*‐test *p* = 0.0018, Figure 3D1). Overall, the results obtained suggest that Mct4 is the main lactate transporter of hypothalamic astrocytes in vitro and in vivo.

### 3.3 Pkm2 is a Regulator of *Slc16a3* (Mct4) mRNA Expression and Lactate Release in Hypothalamic Astrocytes

**FIGURE 4:**
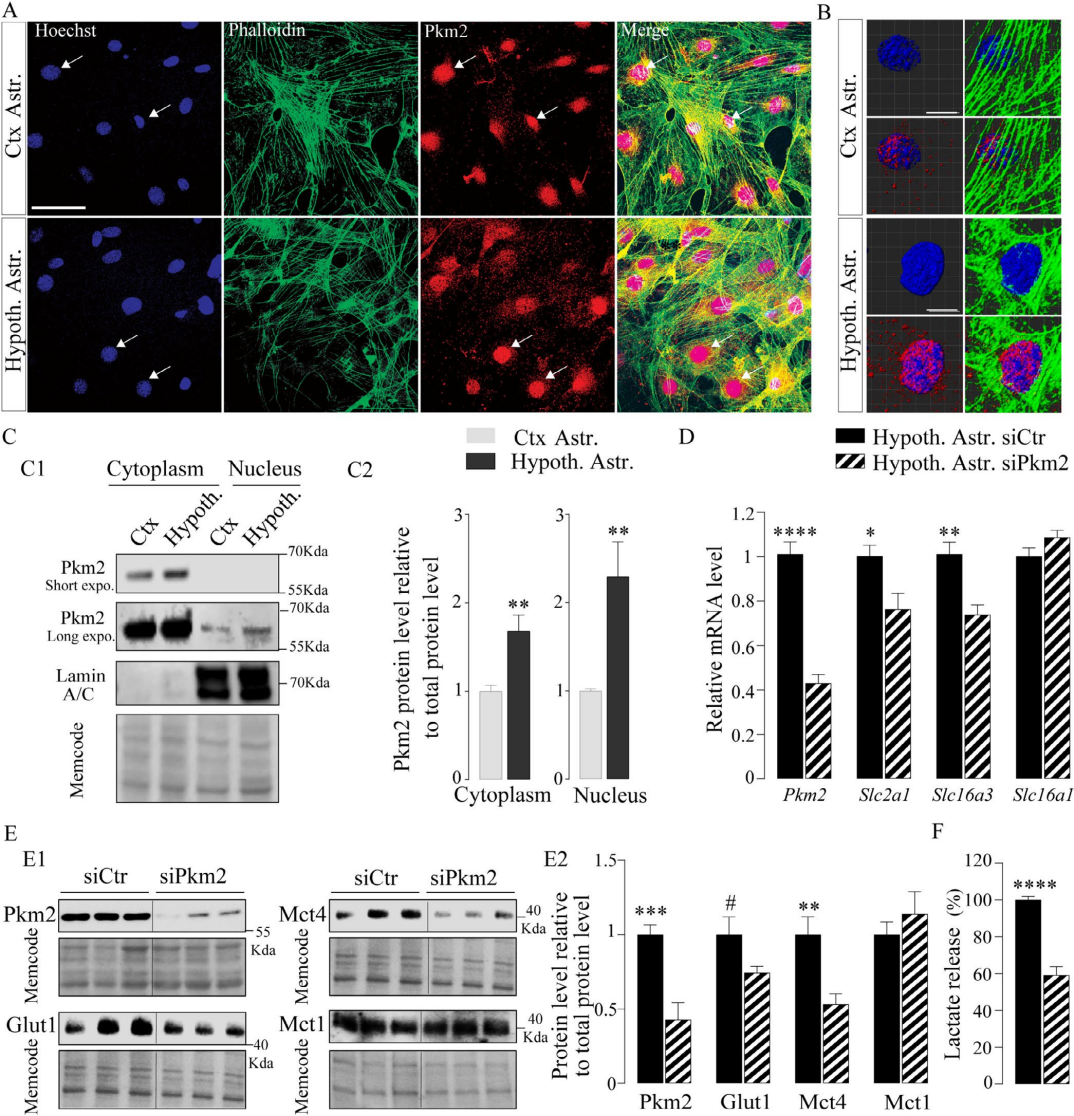
Role of Pkm2 as transcriptional regulator of *Slc16a3* (Mct4) as well as regulator of glycolysis in cultured hypothalamic astrocytes. A–B. Immunofluorescence photomicrographs showing actin using phalloidin (green, cytoskeleton) and Pkm2 immunoreactivity (red) of cultured cortical (top panel) and hypothalamic (bottom panel) astrocytes at low magnification (A) and 3D high magnification (B). Nuclei were stained with Hoechst (blue) C. Representative western blot (C1) and quantification (C2) of Pkm2 protein content in the cytoplasm and nuclei from fractionated cortical and hypothalamic astrocytes (*N* = 4, *n* = 1–3). Lamin A/C is used as nucleus marker D‐E. RT‐qPCR analysis of Pkm2 (*N* = 3, *n* = 3), Slc2a1 (*N* = 1, *n* = 3), Slc16a3 (*N* = 3, *n* = 3), and Slc16a1 (*N* = 1, *n* = 3) mRNA expression in hypothalamic astrocytes in control condition (siCtr) or after silencing of Pkm2 (siPKM2, 48 h, 50 nM) E. Representative western blot (E1) and quantification (E2) of Pkm2 (72 h, *N* = 4, *n* = 3), Glut1 (96 h, *N* = 1, *n* = 3), Mct4 (96 h, *N* = 4, *n* = 3) and Mct1(96 h, *N* = 2, *n* = 3) protein content in hypothalamic astrocytes in siCtr or siPkm2 conditions. Glut1 and Mct4 were probed on the same membrane and share the same MemCode staining. F. Lactate release by hypothalamic astrocytes in siCtr or siPkm2 conditions (96 h, *N* = 4, *n* = 3–6). Statistical analysis was performed using unpaired *t*‐test or Mann–Whitney test. #*p* ≤ 0.1, *p < 0.05; **p < 0.01; ***p < 0.001; ****p < 0.0001. Values indicate means ± SEM. Scale bars (A) 50 μm and (B) 10 μm.

The pyruvate kinase muscle isozyme M2 (Pkm2), which is associated with highly glycolytic cells such as tumor cells, was shown to be a crucial mediator of aerobic glycolysis (Yang and Lu 2013). The Pkm2 isoform, unlike Pkm1, is not only a cytoplasmic key enzyme involved in pyruvate production from PEP (Figure 2F) but also promotes the transcription of genes or the activation of proteins required for aerobic glycolysis (Yang et al. 2012). Egfr translocates Pkm2 to the nucleus, resulting in the upregulation of transcription and expression of Glut1 and Ldha in cancer cells (Yang et al. 2012). Interestingly, astrocytes have a more important basal expression of the Pkm2 isoform than other nontumorigenic cells (Lee et al. 2022), an observation particularly true for hypothalamic astrocytes (Figure 2H). To determine if Pkm2 in astrocytes contributes significantly to their basal metabolic phenotype by acting in the cytoplasm or the nuclei, the intracellular localization of Pkm2 was investigated in hypothalamic and cortical astrocytes in vitro (Figure 4A–C). Pkm2 immunoreactivity (red) was detected in cell bodies (cytoskeleton was stained using phalloidin, green) but also quite intensely in nuclei (arrows, Hoechst staining, blue) of both cortical and hypothalamic astrocytes (Figure 4A,B). To quantify a putative difference in Pkm2 content between astrocytes from both areas, western blots were performed on the cytoplasmic and nuclear fractions (Figure 4C). Pkm2 was approximately two times more abundant in both the cytoplasm (1 ± 0.06 vs. 1.68 ± 0.18, unpaired *t‐*test *p* = 0.002) and nuclei (1 ± 0.02 vs. 2.29 ± 0.39, unpaired *t‐*test *p* = 0.0039) from hypothalamic astrocytes compared to cortical astrocytes (Figure 4C2), suggesting that Pkm2, via the transcriptional regulation of genes involved in aerobic glycolysis, could explain the more pronounced glycolytic profile of hypothalamic astrocytes. Downregulation of *Pkm2* using a siRNA approach in hypothalamic astrocytes (−58.1% ± 6.7%, unpaired *t*‐test, *p* < 0.0001, Figure 4D) caused a decrease in mRNA expression of *Slc16a3* (Mct4, unpaired *t*‐test, *p* = 0.0012, Figure 4D). No effect was observed on *Slc16a1* expression (unpaired *t*‐test, *p* = 0.16, Figure 4D) while a decrease could be detected for *Slc2a1* (Glut1, unpaired *t‐*test, *p* = 0.05, Figure 4D), known to be modulated by Pkm2 expression in other cell types (Yang et al. 2012). At the protein level, siPkm2 transfection induced, as expected, a decrease in Pkm2 abundance (−57.2% ± 13%, unpaired *t*‐test, *p* = 0.0002 Figure 4E) but also a significant decrease in the Mct4 content (unpaired *t*‐test, *p* = 0.002, Figure 4E) without any significant effect on Mct1 production (unpaired *t*‐test, *p* = 0.44, Figure 4E). A decreasing trend was observed in Glut1 protein content (unpaired *t*‐test, *p* = 0.1, Figure 4E). Hypothalamic astrocytes transfected with siPkm2 exhibited a reduction in lactate accumulation in the culture medium (−40.8% ± 4.9%, unpaired *t*‐test *p* < 0.0001, Figure 4F), indicating that a partial knockdown of Pkm2 induced a decrease in lactate production and/or release by/from hypothalamic astrocytes.

Thus, Pkm2 appears as a regulator of *Slc16a3* (Mct4) expression and a crucial mediator of aerobic glycolysis in hypothalamic astrocytes. High production and significant nuclear localization of Pkm2 in hypothalamic astrocytes are likely key for the prominent glycolytic phenotype of these cells.

### 3.4 Glutamate Stimulates Aerobic Glycolysis in Cortical but not Hypothalamic Astrocytes

Both the cortex and the hypothalamus possess glutamatergic as well as GABAergic neurons, but their relative abundance might be different between the two regions. Indeed, acquisition of ^1^H‐NMR spectra of cortical or hypothalamic areas (Figure S3A1,A2) indicated that the glutamate/GABA content ratio was significantly lower in the hypothalamus compared to the cortex (Wilcoxon matched‐pair test, *p* = 0.0039, Figure S3B3). The GABA content in the hypothalamus was not significantly higher, but the glutamate content in the hypothalamus was significantly lower than in the cortex (0.12 ± 0.08 Wilcoxon matched‐pair test *p* = 0.2 and −0.40 ± 0.19 Wilcoxon matched‐pair test *p* = 0.039, respectively, Figure S3B1,B2). Considering the key role of glutamatergic activity on brain glucose utilization involving astrocytes (Voutsinos‐Porche et al. 2003; Zimmer et al. 2017), the effect of glutamate on glucose metabolism was tested and compared between cortical and hypothalamic astrocytes in vitro.

**FIGURE 5:**
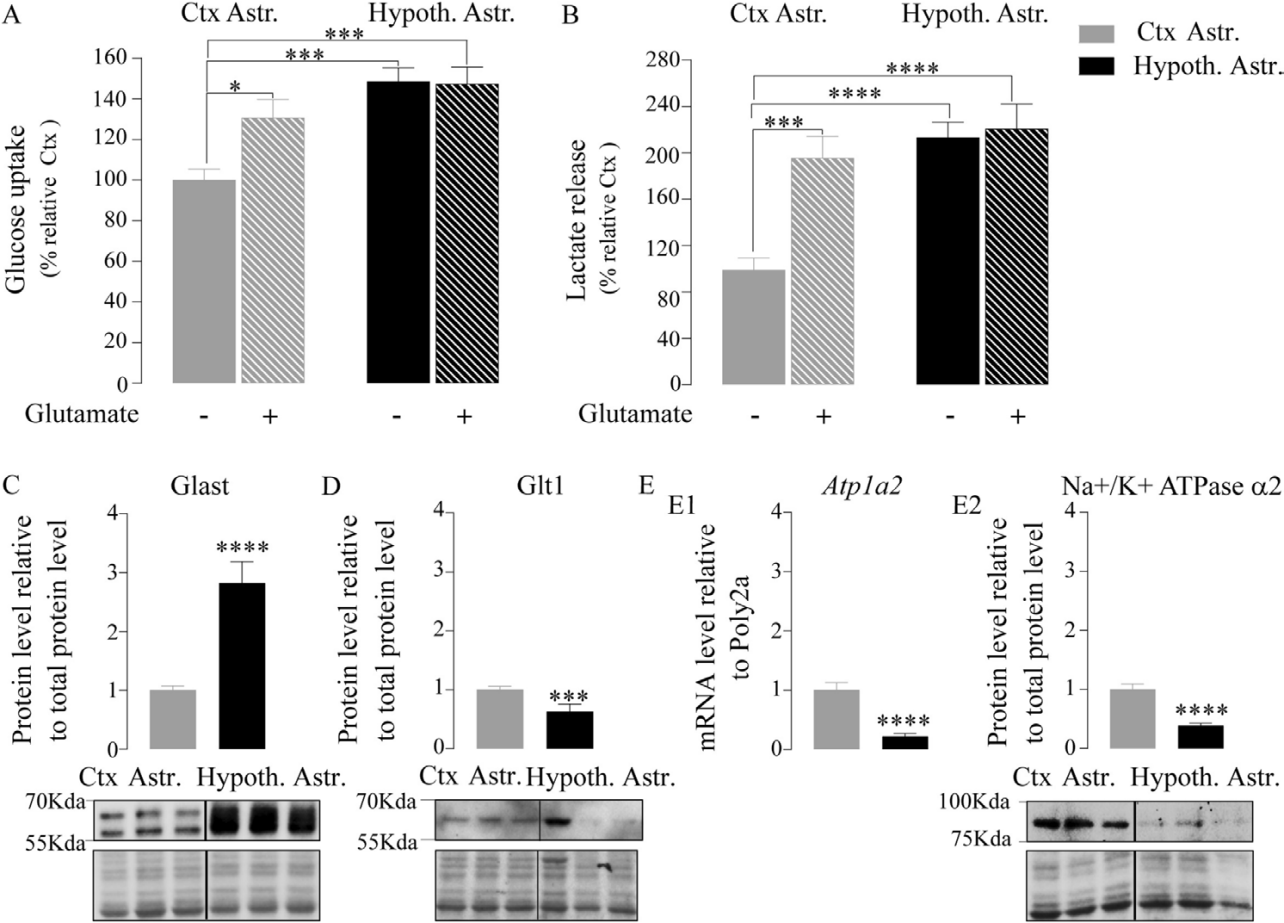
Effect of glutamate on aerobic glycolysis in both cortical and hypothalamic astrocytes. A. ^3^H‐2DG uptake by cortical (gray bar) and hypothalamic (black bar) astrocytes in absence (full bar) or presence (hatched bar) of glutamate (200 μM) after 20 min of incubation (*N* = 4, *n* = 2–3) B. Lactate release by cortical and hypothalamic astrocytes in absence or presence of glutamate (200 μM) after 30 min of incubation (*N* = 3, *n* = 3–6) C‐D. Representative western blot and quantification of glutamate transporter Glast (C) and Glt1 (D) protein expression in cortical and hypothalamic astrocytes (*N* = 3, *n* = 3–6) E. mRNA expression levels (E1, *N* = 4, *n* = 3–6) and protein expression levels with a representative western blot (E2, *N* = 5, *n* = 3–6) of the α_2_ subunit of the Na^+^/K^+^ ATPase pump (*Atp1a2*) in cortical and hypothalamic astrocytes. Statistical analysis was performed using two‐way ANOVA followed by Tukey's multiple comparisons. For simple comparison, unpaired *t*‐test or Mann–Whitney test was performed. **p < 0.01; ***p < 0.001; ****p < 0.0001. Values indicate means ± SEM. See also Figure S3.

Glutamate exposure induced an increase of glucose uptake (+30.6% ± 10.8%, two‐way ANOVA cell types *p* = 0.0002; interaction *p* = 0.04; glutamate stimulation *p* = 0.06, Tukey's multiple comparisons *p* = 0.03, Figure 5A) and lactate release (+96.27% ± 21.34%, two‐way ANOVA cell types *p* = 0.0001; interaction *p* = 0.01; glutamate stimulation *p* = 0.0031; Tukey's multiple comparisons *p* = 0.001, Figure 5B) in cortical astrocytes, as previously demonstrated (Pellerin and Magistretti 1994), but it had no effect on hypothalamic astrocytes (Tukey's multiple comparisons *p* = 0.99 and *p* = 0.98, Figure 5A,B). Interestingly, the levels of glucose uptake and lactate release reached in glutamate‐stimulated cortical astrocytes were equivalent to those of hypothalamic astrocytes under basal (unstimulated) conditions (respectively, 130.6 ± 9.0 vs. 148.5 ± 6.7, *p* = 0.34, and 195.4 ± 18.8 vs. 213.3 ± 13.1, *p* = 0.86). In order to determine if the difference in glycolytic response upon exposure to glutamate depends on glutamate transport capacity, the level of expression of glutamate transporters was investigated in both cortical and hypothalamic astrocytes. Cultured astrocytes from both regions presented a strong expression of the transporter Glast (Figure 5C) and a weak expression of the transporter Glt1 (Figure 5D). But compared to cortical astrocytes, hypothalamic astrocytes exhibited significantly higher levels of the transporter Glast (1.82 ± 0.37 times more, unpaired *t*‐test *p* < 0.0001) and lower levels of the transporter Glt1 (−0.37 ± 0.13 times less, Mann–Whitney test *p* = 0.0008), suggesting that glutamate transport capacity cannot explain the difference in glycolytic response to glutamate. The α_2_ subunit of the Na^+^/K^+^ ATPase is involved in the mechanism to activate aerobic glycolysis in response to glutamate in cortical astrocytes (Pellerin and Magistretti 1997). qPCR and Western blot analysis revealed a lower level of *Atp1a2* mRNA expression (−0.78 ± 0.13, Mann–Whitney test *p* < 0.0001, Figure 5E1) and Na^+^/K^+^ ATPase α_2_ protein content (−0.61 ± 0.1, Mann–Whitney test p < 0.0001, Figure 5E2) in hypothalamic astrocytes compared to cortical astrocytes. These data suggest that the lack of stimulation of glycolysis by glutamate in hypothalamic astrocytes could be explained by the low expression of the Na^+^/K^+^ ATPase α_2_ isoform.

### 3.5 Unlike Cortical Astrocytes, Hypothalamic Astrocytes Modulate Their Lactate Production According to the Extracellular Glucose Concentration in an AMPK‐Dependent Manner

The hypothalamus is a major glucose‐sensing area that participates in the maintenance of whole‐body energy homeostasis. It has been suggested that hypothalamic astrocytes might be sensitive to variations in glucose concentration and participate in the glucose sensing mechanism (Leloup et al. 2016). Because hypothalamic astrocytes exhibited a prominent glycolytic phenotype, the impact of glucose concentration variations on lactate production by these cells was investigated in vitro.

**FIGURE 6:**
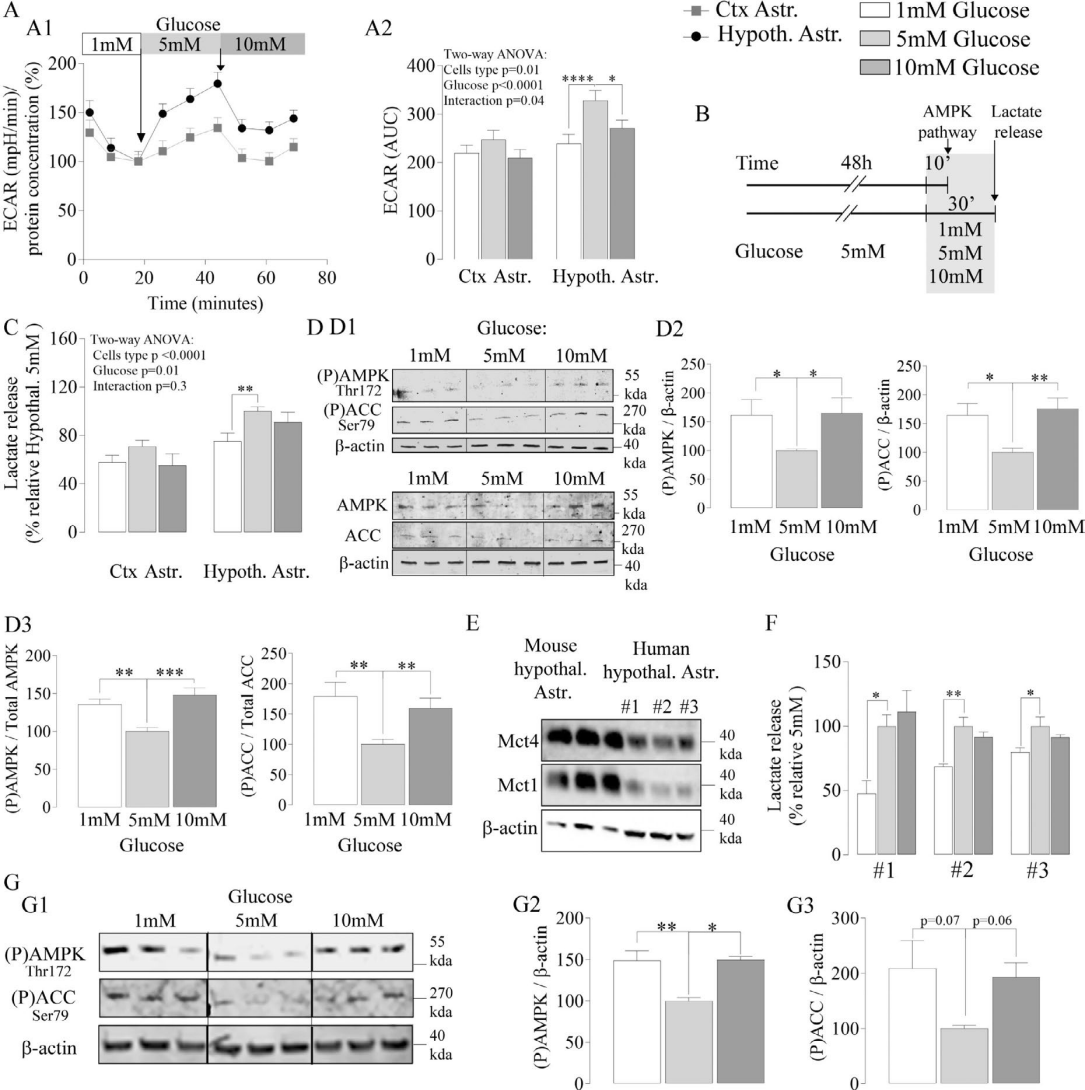
Critical role of AMPK in the glucose‐dependent glycolytic response of hypothalamic astrocytes. A. ECAR profiles of mouse astrocytes from cerebral cortex (gray outline) and hypothalamus (black outline) at 1 mM glucose and after adding 5 and 10 mM glucose (*N* = 2, *n* = 10–11) (A1) and AUC of ECAR measurements from cortical and hypothalamic astrocytes with 1 mM glucose (white bar) and after adding 5 mM (pale gray bar) and 10 mM (dark gray bar) of glucose (A2). B. Protocol to assess the role of AMPK on glucose‐dependent glycolytic response of hypothalamic and cortical astrocytes from mice and humans. C. Quantification of lactate release as a function of the extracellular glucose concentration by hypothalamic (*N* = 3, *n* = 3–6) and cortical astrocytes (*N* = 2, *n* = 3–6) from mice. D. Representative Western blots (D1) and quantification of phosphorylated AMPK/β‐actin and phosphorylated ACC/β‐actin ratios (D2) as well as quantification of phosphorylated AMPK/total AMPK and phosphorylated ACC/total ACC ratios (D3) in mouse hypothalamic astrocytes following variations in extracellular glucose concentration (*N* = 4, *n* = 2–3). For D3, each signal was first normalized to β‐actin from the same membrane prior to calculating the phospho/total protein ratios. E. Representative western blots of Mct1 and Mct4 content in hypothalamic astrocytes from mouse and from three human individuals (#1, #2, #3) cultured in 5 mM glucose. F. Lactate release by human hypothalamic astrocytes at different extracellular glucose concentrations (*n* = 3) G. Representative western blots (G1) and quantitative analysis of phosphorylated AMPK/β‐actin and ACC/β‐actin (G3) of human hypothalamic astrocytes at different extracellular glucose concentrations (*N* = 3, *n* = 3 except for 10 mM, *n* = 3 from 1 human culture). Statistical analysis was performed using one‐way ANOVA followed by Dunnett's multiple comparisons test, Kruskal–Wallis ANOVA followed by Dunn's multiple comparisons test, or two‐way ANOVA followed by Tukey's or Sidak's multiple comparisons tests. *p < 0.05; **p < 0.01; ****p < 0.0001. Values indicate means ± SEM. See also Figure S4.

Cortical and hypothalamic astrocytes were maintained in 5 mM glucose (Figure 6B) to reflect the normal physiological concentration range for the hypothalamus (Levin 2007). Astrocytes were exposed to a final glucose concentration of 1 mM (to mimic hypothalamic hypoglycemia) or to 10 mM glucose (to mimic hypothalamic hyperglycemia). We first explored the effect of glucose concentration variations on the glycolytic profile of both types of astrocytes, using the Seahorse flux analyzer (Figure 6A). At 1 mM glucose, ECAR decreased over time similarly between hypothalamic and cortical astrocytes to reach the same level (white block, Figure 6A1), suggesting that astrocytes from both areas have the same glycolytic rate at that concentration. Injection of 5 mM glucose increased glycolysis to a greater extent in hypothalamic astrocytes (black line, white stripe: 100% ± 10.2%, light gray stripe: 179% ± 11.5%, Figure 6A1) compared to cortical astrocytes (gray line, white stripe: 100% ± 7.2%, light gray stripe: 134% ± 0.2%, Figure 6A1), confirming our previous data (Figure 2A). Injection of 10 mM glucose induced a fast decrease of ECAR in both cell types (dark gray stripe, Ctx. Astr.: 103.6% ± 9.2%; Hypoth. Astr.: 133.9% ± 9.1%, Figure 6A1), suggesting a stress response of astrocytes to high glucose concentration. Twenty minutes after 10 mM glucose injection (corresponding to time point 70 min), ECAR started to recover in both cell types (end of dark gray stripe, Ctx Astr.: 114.7% ± 8.5%; Hypoth. Astr.: 143.9% ± 8.3%, Figure 6A1). The area under the curve analysis of the rate of acidification of the culture medium (ECAR) during a 20‐min period (which corresponds to the amount of lactate released during 20 min) for cortical astrocytes was similar regardless of the glucose concentration (1 mM: 219 ± 16.56, 5 mM: 247.1 ± 19.7, 10 mM: 209.6 ± 17.13, two‐way ANOVA, Sidak's multiple comparisons tests, 1–5 mM: *p* = 0.50, 5–10 mM: *p* = 0.18, Figure 6A2). In contrast, AUCs of ECAR for hypothalamic astrocytes were significantly lower at 1 mM glucose (239% ± 19%, two‐way ANOVA Sidak's multiple comparisons tests, *p* < 0.0001, Figure 6A2) and 10 mM glucose (270.9% ± 16.92%, two‐way ANOVA, Sidak's multiple comparisons tests, *p* = 0.01, Figure 6A2) compared to 5 mM glucose (328% ± 21%). These first observations suggest that hypothalamic astrocytes are more sensitive to glucose concentration variations compared to cortical astrocytes.

To mimic physiological conditions, astrocytes maintained at 5 mM of glucose were directly exposed to 1 mM of glucose or 10 mM of glucose (Figure 6B) before measuring lactate release from both cell types. No significant changes in lactate concentration were observed in the culture medium from cortical astrocytes exposed for 30 min to 1, 5, or 10 mM glucose (two‐way ANOVA, Sidak's multiple comparisons test, 1–5 mM *p* = 0.37, 5–10 mM *p* = 0.24, Figure 6C). Hypothalamic astrocytes exposed to 1 mM glucose had a significantly reduced level of released lactate compared to cells maintained in 5 mM glucose (75% ± 6.8% vs. 100% ± 3.5%, two‐way ANOVA, Sidak's multiple comparisons test, *p* = 0.009, Figure 6C). Hypothalamic astrocytes already presented a significant decrease of released lactate levels as glucose levels were reduced from 5 to 4 mM glucose and 3 mM (one‐way ANOVA *p* < 0.0001, Dunnett's multiple comparisons test 5–4 mM: *p* = 0.0003, 5–3 mM: p = 0.0008, Figure S4A) and further down to 1 mM glucose (one‐way ANOVA, Dunnett's multiple comparisons test *p* < 0.0001, Figure S4A). In parallel, increasing glucose concentrations from 5 to 6 mM or 8 mM induced a significant increase in lactate release levels (one‐way ANOVA *p* = 0.008, Dunnett's multiple comparisons test 5–6 mM: *p* = 0.02 and 5–8 mM: *p* = 0.03, respectively, Figure S4B). Interestingly, further increasing the glucose concentration to 10 mM did not enhance lactate release but rather maintained it at the level of the control (5 mM glucose) condition (100.1 ± 9.94 vs. 100 ± 3.8, one‐way ANOVA, Dunnett's multiple comparisons test, *p* > 0.99, Figure S4B and 90.99 ± 8.1 vs. 100 ± 3.5 two‐way ANOVA, Sidak's multiple comparisons test, *p* = 0.49 Figure 6C). We also explored the glycolytic response of hypothalamic astrocytes by gradually increasing glucose levels from 1 to 2, 3, 4, or 5 mM using the Seahorse flux analyzer (Figure S4C). The AUCs of ECAR for hypothalamic astrocytes significantly increased from 1 to 4 mM glucose and 1–5 mM glucose (Two‐way ANOVA, Sidak's multiple comparisons test 1–4 mM: *p* = 0.03, 1–5 mM: *p* < 0.0001, Figure S4C2), demonstrating that hypothalamic astrocytes respond to both increases and decreases in glucose levels within physiological ranges (1–5 mM).

Thus, both analyses (lactate concentration measurement and ECAR) suggest that glycolysis in hypothalamic astrocytes is sensitive to changes in the extracellular glucose concentration, while it is not the case for cortical astrocytes. These observations raised the question of the identity of the molecular component(s) responsible for this hypothalamic glucose‐sensing response.

The enzyme AMP‐activated protein kinase (AMPK) is a major sensor of the cellular energetic status in various tissues, including the central nervous system and, more specifically, in astrocytes (Muraleedharan and Dasgupta 2022). An increase in the AMP/ATP ratio occurs when the glycolytic flux rate decreases, but an increase in calcium (Ca^2+^)‐induced CaMKII stimulation can also cause AMPK phosphorylation and its activation (Muraleedharan and Dasgupta 2022). The impact of extracellular glucose concentration variations not only on AMPK phosphorylation but also on acetyl‐CoA carboxylase (ACC) phosphorylation (a target of AMPK) was investigated in hypothalamic astrocytes (Figure 6D). After 10 min, hypothalamic astrocytes placed in 1 mM glucose presented a significant increase of AMPK and ACC phosphorylation levels compared to 5 mM glucose (Kruskal–Wallis ANOVA *p* < 0.001, Dunn's multiple comparisons test AMPK/β‐actin *p* = 0.019, AMPK/total AMPK *p* = 0.007, ACC/β‐actin *p* = 0.017 and ACC/total ACC *p* = 0.002, Figure 6D), most likely due to a decrease of ATP production. Phosphorylation of AMPK increases with smaller variations in glucose concentration, reaching significance at 3 mM (one‐way ANOVA *p* = 0.023, Dunnett's multiple comparisons test *p* = 0.028, Figure S4D) and 2 mM (one‐way ANOVA p = 0.023, Dunnett's multiple comparisons test *p* = 0.043, Figure S4D), which correlates with the observed decrease in lactate concentration (Figure S4A). Surprisingly, the same response was observed after being placed in 10 mM glucose (Kruskal–Wallis ANOVA *p* < 0.001, Dunn's multiple comparisons test, AMPK/β‐actin *p* = 0.03 AMPK/total AMPK *p* = 0.0006, ACC/β‐actin p = 0.007, and ACC/Total ACC *p* = 0.0049, Figure 6D). In this case, the effect might be due to stress‐induced intracellular Ca^2+^ rise, as the injection of 10 mM glucose induced a fast decrease of ECAR (Figure 6A1). Indeed, it is known that intracellular stress induces Ca^2+^ rise in the hypothalamus as well as in astrocytes themselves (Gyengesi et al. 2012; Murphy‐Royal et al. 2020) while Ca^2+^ release increases AMPK activity in the hypothalamus (Lòpez et al. 2016). As compared to the control group (5 mM glucose), hypothalamic astrocytes treated with 10 mM glucose showed a rapid and significant peak of cytosolic Ca^2+^, with a maintained plateau that progressively decreased over time (*T*‐test *p* = 0.0002, Figure S4E).

A pharmacological approach was used to demonstrate that AMPK activation is responsible for the glycolytic response of hypothalamic astrocytes to changes in extracellular glucose concentration (Figure S4F). The AMPK inhibitor Compound C not only prevented the decrease of lactate release normally observed at 1 and 10 mM glucose but caused an enhancement above levels normally observed at 5 mM glucose (unpaired *t*‐test *p* = 0.011 and *p* = 0.0007, Figure S4F). In contrast, incubation with the AMPK activator AICAR induced a decrease in lactate release at 5 mM glucose (unpaired *t*‐test *p* = 0.005, Figure S4F). These data suggest that a change from 5 to 1 mM or from 5 to 10 mM glucose activates the AMPK sensor in hypothalamic astrocytes and inhibits glycolysis via, respectively, a putative increase of the AMP/ATP ratio and an elevation of intracellular calcium levels.

Considering the implications of a putative glucose‐sensing role of hypothalamic astrocytes in human pathophysiology (e.g., in eating disorders and obesity), it became important to verify if the current findings can be confirmed in human hypothalamic astrocytes (Figure 6E–G). Indeed, human hypothalamic astrocytes, like those from mice, were found to express the lactate transporters MCT1 and MCT4 (Figure 6E). A decrease in the extracellular glucose concentration from 5 to 1 mM induced a decrease in lactate production in cultures from three different individuals (one‐way ANOVA, Dunnett's multiple comparisons test #1 *p* = 0.04, #2 *p* = 0.006, #3 p = 0.04, Figure 6F), as observed in rodents (Figure 6C). An increase in the extracellular glucose concentration from 5 to 10 mM induced a small, but not significant, decrease in lactate release in cultures from individuals #2 and #3 (−8.3 ± 7.9 and −8.7 ± 7.4, one‐way ANOVA Dunnett's multiple comparisons test *p* = 0.4 and *p* = 0.38, respectively, Figure 6F). Glucose concentration variations from 5 to 1 or 10 mM induced an increase in AMPK phosphorylation (Kruskal–Wallis ANOVA *p* < 0.001, Dunn's multiple comparisons test *p* = 0.002 and p = 0.01 Figure 6G1,G2) as observed above in rodents (Figure 6D). However, ACC phosphorylation did not reach statistical significance, although a trend toward an increase was observed (Kruskal–Wallis ANOVA *p* = 0.02, Dunn's multiple comparisons *p* = 0.07 and *p* = 0.06, Figure 6G1,G3). The response of both lactate release and AMPK phosphorylation levels to changes in glucose concentration is consistent with the existence of a similar glucose‐sensing mechanism taking place in human hypothalamic astrocytes.

## 4 Discussion

Cortical astrocytes are known to exhibit a prominent glycolytic capacity with high glucose utilization and lactate release when compared to cortical neurons (Hertz et al. 1988; Itoh et al. 2003; Walz and Mukerji 1988). Such a glycolytic profile was found in our hands to be even more pronounced in hypothalamic astrocytes compared to cortical astrocytes. Significant heterogeneity has been observed among astrocytes from different brain regions, as demonstrated by single‐cell RNA sequencing (Hasel et al. 2023). Here, we found that the expression levels of the glucose transporter Glut1, the hexokinase isoform Hk2, the pyruvate kinase isoform Pkm2, and the monocarboxylate transporter Mct4 were higher in hypothalamic versus cortical astrocytes. Consistently, the Ldh5/Ldh1 ratio, which favors lactate over pyruvate formation, is higher in hypothalamic astrocytes. Moreover, it was demonstrated that the strong expression of Mct4 was essential for exhibiting this high glycolytic profile in hypothalamic astrocytes, while the other astrocytic lactate transporter, Mct1, was dispensable. It is noteworthy that Mct4 promotes glycolysis better than Mct1 by allowing lactate to be exported despite elevated extracellular lactate levels (Contreras‐Baeza et al. 2019). Interestingly, high expression of both Pkm2 and Mct4 has previously been reported in various cancer cell types, which are well known for their highly glycolytic activity (Pérez‐Escuredo et al. 2016; Zhu et al. 2021). Expression of Pkm2 was identified as the key element to shift cellular metabolism to aerobic glycolysis (known as the Warburg effect) in tumor cells, but also in non‐pathological cells such as astrocytes (Christofk et al. 2008; Lee et al. 2022). Pkm2 expression level is likely the main factor to determine the degree of glycolytic activity, as observed between hypothalamic and cortical astrocytes. It can be concluded that hypothalamic astrocytes appear genetically programmed to exhibit a strong basal glycolytic profile and eventually produce a strong glycolytic response to specific challenges.

### 4.1 Glycolytic Response of Astrocytes to Glutamate

Glutamate and GABA are the most abundant neurotransmitters in the cerebral cortex and are responsible for the excitatory and inhibitory neurotransmission, respectively, in the central nervous system. They are involved in the control of several fundamental cerebral functions, including neuro‐energetics, and their balance is important for normal brain operation. Using NMR spectroscopy, we measured a significant difference in glutamate and glutamate/GABA content between the cerebral cortex and the hypothalamus in the rat brain. Indeed, using a different technique (HPLC), it was previously observed in rodents that the glutamate/GABA content ratio was different between the cortex and the hypothalamus (Franco‐Pérez et al. 2020). It was also shown that GABAergic TCA cycle flux and GABAergic neurotransmission were significantly higher in the thalamus–hypothalamus, while the glutamatergic TCA and the glutamatergic neurotransmitter cycle rates were significantly higher in the mouse cerebral cortex (Tiwari et al. 2013). These data, together with ours, suggest that the energetics of excitatory and inhibitory neurotransmission might be distinct across the brain, as proposed by Tiwari et al. (2013). Moreover, it might be regulated by distinct mechanisms and/or at different levels in different brain regions, implicating astrocytes, as suggested for cortical structures (Voutsinos‐Porche et al. 2003; Zimmer et al. 2017). Indeed, it was shown that both K^+^ (Bittner et al. 2011) and NH_4_^+^ (Lerchundi et al. 2015) can also stimulate glycolysis and lactate release by cortical astrocytes.

It was previously shown that glutamate (but not GABA) stimulates aerobic glycolysis in astrocytes from the cortex, the hippocampus, and the cerebellum (Chatton et al. 2003; Pellerin and Magistretti 2005). This response was shown to depend on the uptake of glutamate via the Na^+^‐dependent glutamate transporters Glast (Pellerin and Magistretti 1994) or Glt1 (Zimmer et al. 2017). The concomitant Na^+^ entry leads to a specific activation of the Na^+^/K^+^ ATPase α_2_ subunit (Chatton et al. 2003; Pellerin and Magistretti 1997), causing a stimulation of glycolysis and lactate release (Pellerin and Magistretti 1994, 2005). Interestingly, hypothalamic astrocytes do not exhibit any glycolytic response to glutamate. The lack of response does not depend on the level of glutamate transporter expression (and thus putatively on the extent of intracellular Na^+^ concentration elevation) since they even possess a higher level of Glast expression than cortical astrocytes. However, their level of expression of the Na^+^/K^+^ ATPase α_2_ subunit is less than half of the level observed in cortical astrocytes. This characteristic might be responsible for the absence of glycolytic activation upon glutamate exposure. Indeed, it was found that the expression of the Na^+^/K^+^ ATPase α_2_ subunit in combination with the β_1_ subunit makes this isoform more sensitive to physiological elevation in intracellular Na^+^ concentrations (Larsen et al. 2016). Thus, it makes this isoform more prone to be activated by glutamate uptake, which is co‐transported with sodium, leading to the activation of glycolysis. Indeed, it was previously shown that even in the absence of Glast or Glt1, the same level of glycolysis activation can be achieved by activating AMPA receptors and preventing their desensitization, which also leads to an elevation of intracellular Na^+^ levels (Voutsinos‐Porche et al. 2003), demonstrating the dominant role played by the Na^+^/K^+^ ATPase α_2_ subunit. In contrast, cells expressing predominantly isoforms containing the α_1_ subunit would not exhibit such behavior, as we observed with hypothalamic astrocytes. It appears unlikely, then, that hypothalamic astrocytes respond to glutamatergic activity with glutamate‐dependent metabolic neuronal support, as is the case in other brain regions.

### 4.2 Role of Hypothalamic Astrocytes in Metabolic Sensing

The hypothalamus is a structure composed of several nuclei. Some of them play a critical role in the maintenance of whole‐body energy homeostasis. This is the case notably for the ventromedial hypothalamus (VMH) and the lateral hypothalamus (LH), which are involved in the regulation of food intake. In each of these nuclei can be found some specific neuronal populations, which respond to variation in circulating hormones and metabolites. Glycemia is a parameter that is monitored within the hypothalamus and serves as a trigger for counterregulatory responses, which include regulation of food intake. Basal glucose concentration and its evolution after normal feeding in the ventromedial hypothalamus have been determined by microdialysis (de Vries et al. 2003; Mayer et al. 2006). It was found to be in the 0.7–0.94 mM range for the basal concentration and goes up to 4.23 mM after normal feeding in fasted rats. When blood glucose levels fluctuate from pathological hypoglycemia to hyperglycemia, hypothalamic glucose levels range from ~0.2 to 4.5 mM (Routh et al. 2014) and can occasionally reach up to 8–10 mM (Poitry‐Yamate et al. 2009). Two populations of neurons have been identified, which are sensitive to glucose concentration variations in the range 0.1–2.5 mM. These are glucose‐excited (GE) and glucose‐inhibited (GI) neurons, collectively known as glucose‐sensing neurons (Routh et al. 2014). In addition to these neuronal populations, it has been suggested that astrocytes could participate in the detection of changes in glycemia and the associated counterregulation responses (García‐Cáceres et al. 2019; Herrera Moro Chao et al. 2022; Leloup et al. 2016). In this study, we demonstrated that hypothalamic astrocytes are sensitive to a variation of glucose concentrations within a physiological range, causing a modulation of lactate production. Our data are consistent with previous observations obtained in vivo showing that enhanced glucose levels due to feeding or direct perfusion cause an increase in extracellular lactate levels in the ventromedial‐paraventricular hypothalamus as measured by microdialysis (Goucham and Nicolaïdis 1999).

At the mechanistic level, it was previously found that in vivo hypothalamic astrocytes express the glucose transporter Glut2 and the Hexokinase IV or Glucokinase, two important elements that determine the glucose‐sensing capacity of pancreatic β‐cells (Arluison, Quignon, Thorens, et al. 2004; Arluison, Quignon, Nguyen, et al. 2004; Leloup et al. 1994; Millán et al. 2010; Pasula et al. 2023). In our hands, expression of these two isoforms by astrocytes in vitro is not sufficient to account for a glucose‐sensing phenotype. Rather, the energy sensor AMPK turned out to be critical for the capacity of hypothalamic astrocytes to convert changes in glucose concentrations into different lactate production rates in vitro. Energetic stress caused by changes in glucose concentrations induces AMPK activation in hypothalamic astrocyte cultures. Both ATP level reduction and a Ca^2+^ rise cause the phosphorylation of AMPK after exposure to, respectively, low and high levels of glucose. The implication of hypothalamic AMPK in the regulation of food intake and energy balance, including changes in glucose concentrations, has been well documented (Andersson et al. 2004; Minokoshi et al. 2004). Although the role of AMPK in glucose sensing by certain populations of hypothalamic neurons has been reported (Claret et al. 2007; Murphy, Fakira, et al. 2009; Murphy, Fioramonti, et al. 2009), its importance for the mechanism of glucose sensing by astrocytes has not been demonstrated yet. Our data provide clear evidence that AMPK is not only essential for long‐chain fatty acid metabolism and sensing in hypothalamic astrocytes (Taïb et al. 2013) but also regulates lactate production by hypothalamic astrocytes concomitantly with changes in extracellular glucose concentrations.

Lactate is not only a prominent energy substrate for neurons; it was also shown to regulate neuronal excitability via an effect involving K^+^‐ATP channels (Karagiannis et al. 2021). It was previously demonstrated that hypothalamic glucose sensing to regulate blood glycemia requires local conversion of glucose into lactate, followed by its metabolism via pyruvate and activation of K^+^‐ATP channels (Lam et al. 2005). Indeed, there are several reports showing that lactate regulates the activity of different glucose‐sensing neuronal populations in the hypothalamus (Ainscow et al. 2002; Lhomme et al. 2021; Mobbs et al. 2001; Ordenes et al. 2021; Song and Routh 2005). These observations led to the concept that astrocytes could participate in glucose sensing in the hypothalamus via their capacity to release lactate (which would influence the activity of key neuronal populations), although the specific molecular mechanism had remained elusive (Allard et al. 2013; Leloup et al. 2016). Our data now provide an insight into the molecular features that determine the glucose‐sensing capacity of hypothalamic astrocytes. Indeed, these cells are geared to fulfill this role. They constitutively exhibit high glycolytic capacity and maximal glycolytic activity with optimal glucose concentration, which are determined by a high expression and nuclear localization of the isoform Pkm2, and a high expression of the glycolytic enzyme hexokinase 2 and the lactate transporter Mct4. In addition, they respond to changes in glucose concentrations by altering their lactate production, a feature that depends on the intracellular energy sensor AMPK. As lactate levels in their immediate vicinity can alter the activity of various hypothalamic neuronal populations that are key for the regulation of energy homeostasis, such a metabolic cooperation between astrocytes and neurons of the hypothalamus constitutes an important glucose‐sensing mechanism.

## 5 Conclusion

This study shows that hypothalamic astrocytes possess a higher glycolytic metabolism and no sensitivity for glycolysis stimulation by glutamate compared to cortical astrocytes. In contrast, they respond to elevation in glucose concentrations by stimulation of lactate production. These data suggest that hypothalamic astrocytes possess a different metabolic phenotype compared to cortical astrocytes, a characteristic that could explain their particular glucose sensitivity and their participation in hypothalamic glucose‐sensing mechanisms.

## Author Contributions

Conceptualization S.G. and L.P.; Methodology S.G., S.L., N.Z., T.Z., A.‐K.B.‐S.; Investigation S.G. performed all the experiments with the help of S.L., N.Z., T.Z., C.G., T.S., C.R.; A.‐K.B.‐S. performed NMR measurements, and A.S. performed human primary cultures of hypothalamic astrocytes. Data analysis S.G., S.L., N.Z., A.‐K.B.‐S.; writing of original draft S.G. and L.P.; corrected manuscript: S.G., N.Z., S.L., T.Z., C.G., F.A., A.‐K.B.‐S., A.S., L.P.; project administration S.G.; funding acquisition and resources P.B., F.A., A.‐K.B.‐S., A.S., L.P.; project supervision L.P.

## Conflicts of Interest

The authors declare no conflicts of interest.

## Supporting information


**Figure S1.** Metabolic flux analyses and expression of some key glycolytic elements in cortical and hypothalamic astrocytes. A, B. Seahorse flux analysis of extra cellular acidification rate (ECAR (A)) and OCR (B) of cortical and hypothalamic astrocytes before (gray frames, 0.1 mM glucose) and after adding 5 mM glucose, 2DG (50 mM), UK5099 2 μM (mitochondrial pyruvate carrier inhibitor), anti‐antimycin A, and rotenone 1 μM (complex III and I inhibitors, respectively) (*n* = 5). C, E–H. Representative western blots for Glut1, Hk2, Pkm2, Mct1, and Mct4 content in cortical and hypothalamic astrocytes. D. RT‐qPCR (top panel) and western blots (bottom panel) analysis for Glut2 transporters (*N* = 1–3, *n* = 4–6) and some glycolytic enzymes (Hk1: *N* = 3, *n* = 3–5; Gk: *N* = 1–2, *n* = 3–6; Pkm1: *N* = 3, *n* = 4–6; Ldh5: *N* = 3–5, *n* = 3–6; and Ldh1: *N* = 3–5, *n* = 3–6) in cortical and hypothalamic astrocytes. Statistical analysis was performed using unpaired *t*‐test or Mann–Whitney test. ***p < 0.001. Values indicate means ± SEM.

**Figure S2.** Cell sorting of hGFAP‐eCFP cells from the cortex and the hypothalamus by FACS, as well as schematic representations of the strategy to generate conditional transgenic MCT1 and MCT4 KO mouse lines. A. Dot plot illustrating sorting gates for [eCFP^+^] cells (Pos) and [eCFP^−^] cells (Neg) isolated from cortex (left plot) and hypothalamus (right plot) from hGFAP‐eCFP mice. B–C. Schematic representation of the strategy used to generate the alleles of the conditional transgenic mouse lines Mct4 (B, Mct4^fl/fl^) and Mct1 (C, Mct1^fl/fl^).

**Figure S3.** Glutamate and GABA content in the barrel cortex and medio‐basal hypothalamus of adult male rats using HR‐MAS NMR spectroscopy. A. Schematic brain sections illustrating brain biopsies analyzed by HR‐MAS ^1^H‐NMR spectroscopy (A1) and representative NMR spectra of barrel cortex (blue line) and medio‐basal hypothalamus (red line) obtained from the same rat (A2). B. Comparative analysis of glutamate content (Glu, B1), GABA content (B2), and glutamate/GABA ratio (B3) between the barrel cortex (gray bar, blue circle) and the medio‐basal hypothalamus (black bar, red circle) obtained from the same rats (*N* = 9). Statistical analysis was performed using Wilcoxon test. *p < 0.05; **p < 0.01. Values indicate means ± SEM.

**Figure S4.** Effects of glucose concentration variations on lactate release, calcium levels, and AMPK, as well as ACC phosphorylation in mouse hypothalamic astrocytes. A–B. Lactate release by hypothalamic astrocytes after exposure to different extracellular glucose concentrations from 5 to 1 mM (A, *N* = 2, *n* = 3) and from 5 to 10 mM (B, *N* = 1, *n* = 4–6). C. ECAR profiles of mouse astrocytes from hypothalamus at 1 mM glucose (gray background) and after adding glucose to a final concentration of 2, 3, 4, and 5 mM (*N* = 1, *n* = 5 per condition), oligomycin (1 μM) and 2DG (50 mM) (C1) and AUC of ECAR measurements hypothalamic astrocytes with 1 mM glucose (gray background) and after adding glucose (C2). D. Representative western blots and quantification of phosphorylated AMPK/total AMPK ratios of mouse hypothalamic astrocytes (from C57B6J pups) after extracellular glucose concentration variations (*N* = 3, *n* = 2–3). E. (E1) Representative graph of cytosolic Ca^2+^ transients in hypothalamic astrocytes stimulated with 5 or 10 mM glucose. (E2) Quantification of cytosolic Ca^2+^increase in hypothalamic astrocytes in response to 5 mM (pale gray, *N* = 5, *n* = 5–13 cells) or 10 mM (dark gray, *N* = 4, *n* = 5–14 cells) glucose stimulation. F. Lactate release by mouse hypothalamic astrocytes after extracellular glucose concentration variations in the presence or absence of Compound C (Comp. C, 1 mM) or AICAR (5 mM) (*N* = 1, *n* = 3). Statistical analysis was performed using unpaired *t*‐test or Mann–Whitney test for simple comparison and one‐way ANOVA followed by Dunnett’s multiple‐comparisons test with the control. *p < 0.05; **p < 0.01; ***p < 0.001. Values indicate means ± SEM.

Data S1.

## Data Availability

The data that support the findings of this study are available on request from the corresponding author.
