# Supplementary figures and images for: Hypothalamic Astrocytes Exhibit Glycolytic Features Making Them Prone for Glucose Sensing
Source: Glia. 2025 Jul 24;73(11):2253–72. doi: 10.1002/glia.70066 (PMC12436996; doi:10.1002/glia.70066)

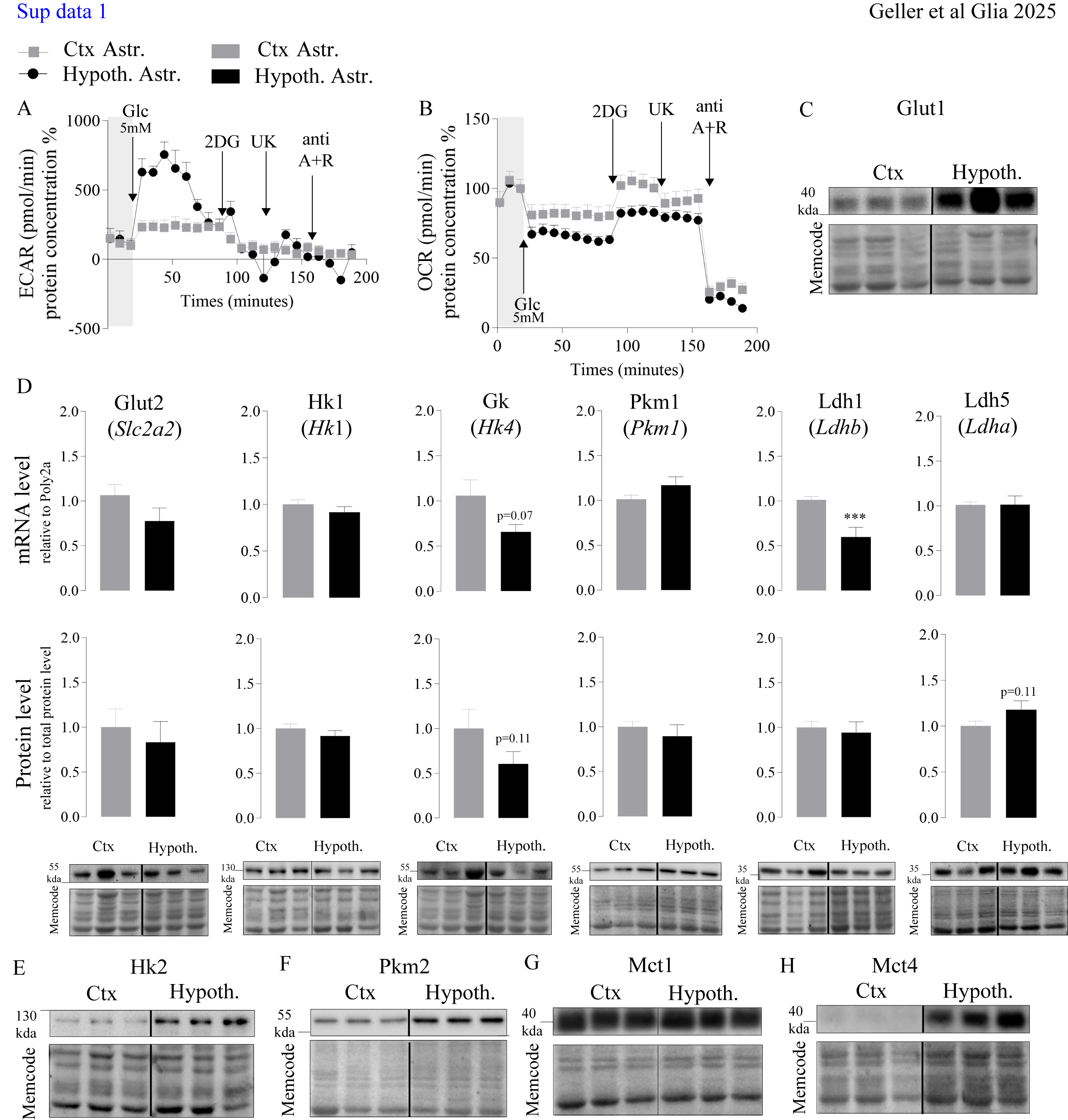

Supplement: Supplementary file 1 — Figure S1. Metabolic flux analyses and expression of some key glycolytic elements in cortical and hypothalamic astrocytes. A, B. Seahorse flux analysis of extra cellular acidification rate (ECAR (A)) and OCR (B) of cortical and hypothalamic astrocytes before (gray frames, 0.1 mM glucose) and after adding 5 mM glucose, 2DG (50 mM), UK5099 2 μM (mitochondrial pyruvate carrier inhibitor), anti‐antimycin A, and rotenone 1 μM (complex III and I inhibitors, respectively) (n = 5). C, E–H. Representative western blots for Glut1, Hk2, Pkm2, Mct1, and Mct4 content in cortical and hypothalamic astrocytes. D. RT‐qPCR (top panel) and western blots (bottom panel) analysis for Glut2 transporters (N = 1–3, n = 4–6) and some glycolytic enzymes (Hk1: N = 3, n = 3–5; Gk: N = 1–2, n = 3–6; Pkm1: N = 3, n = 4–6; Ldh5: N = 3–5, n = 3–6; and Ldh1: N = 3–5, n = 3–6) in cortical and hypothalamic astrocytes. Statistical analysis was performed using unpaired t‐test or Mann–Whitney test. ***p < 0.001. Values indicate means ± SEM. [file GLIA-73-2253-s001.tif]

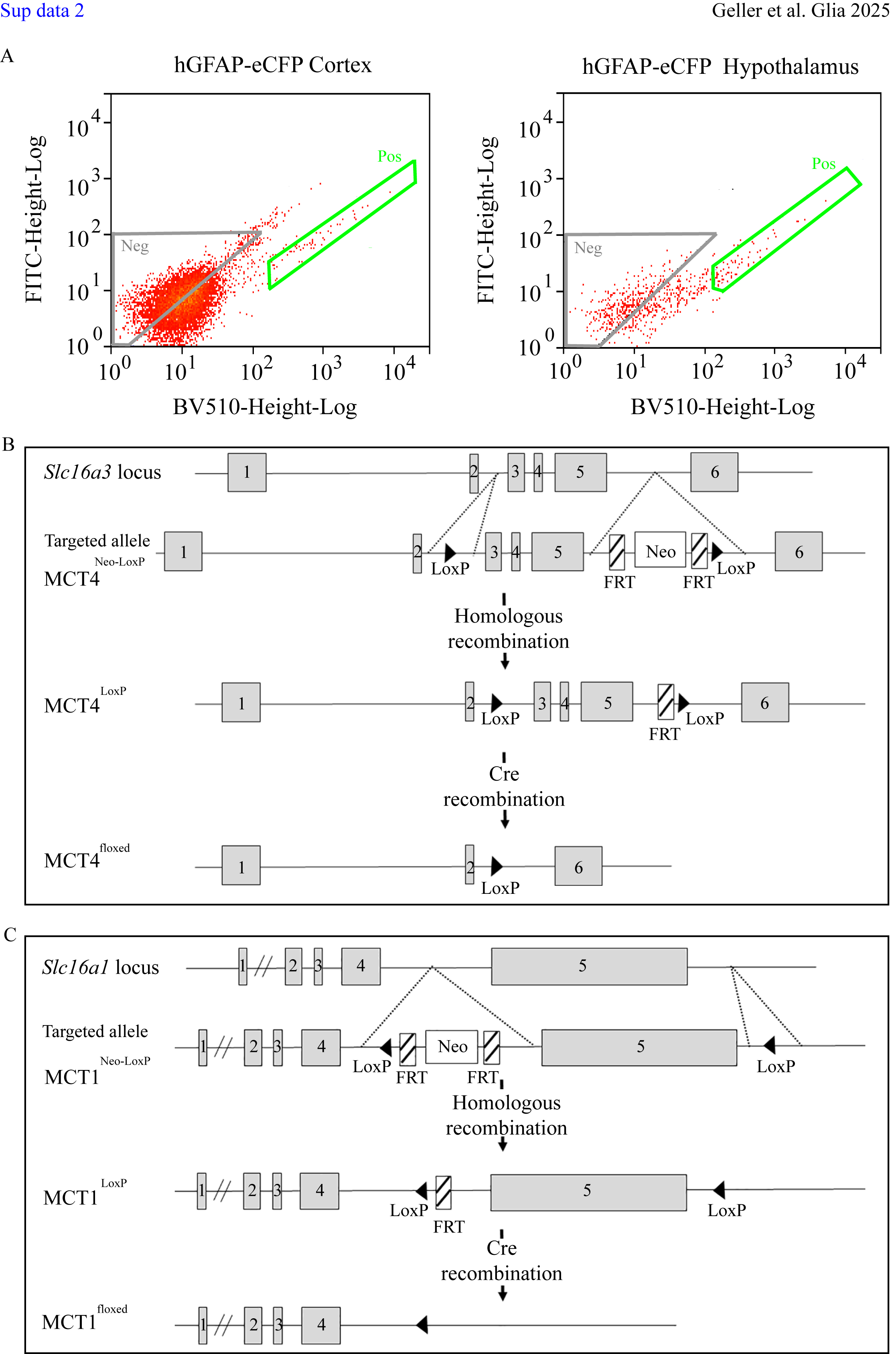

Supplement: Supplementary file 2 — Figure S2. Cell sorting of hGFAP‐eCFP cells from the cortex and the hypothalamus by FACS, as well as schematic representations of the strategy to generate conditional transgenic MCT1 and MCT4 KO mouse lines. A. Dot plot illustrating sorting gates for [eCFP+] cells (Pos) and [eCFP−] cells (Neg) isolated from cortex (left plot) and hypothalamus (right plot) from hGFAP‐eCFP mice. B–C. Schematic representation of the strategy used to generate the alleles of the conditional transgenic mouse lines Mct4 (B, Mct4fl/fl) and Mct1 (C, Mct1fl/fl). [file GLIA-73-2253-s005.tif]

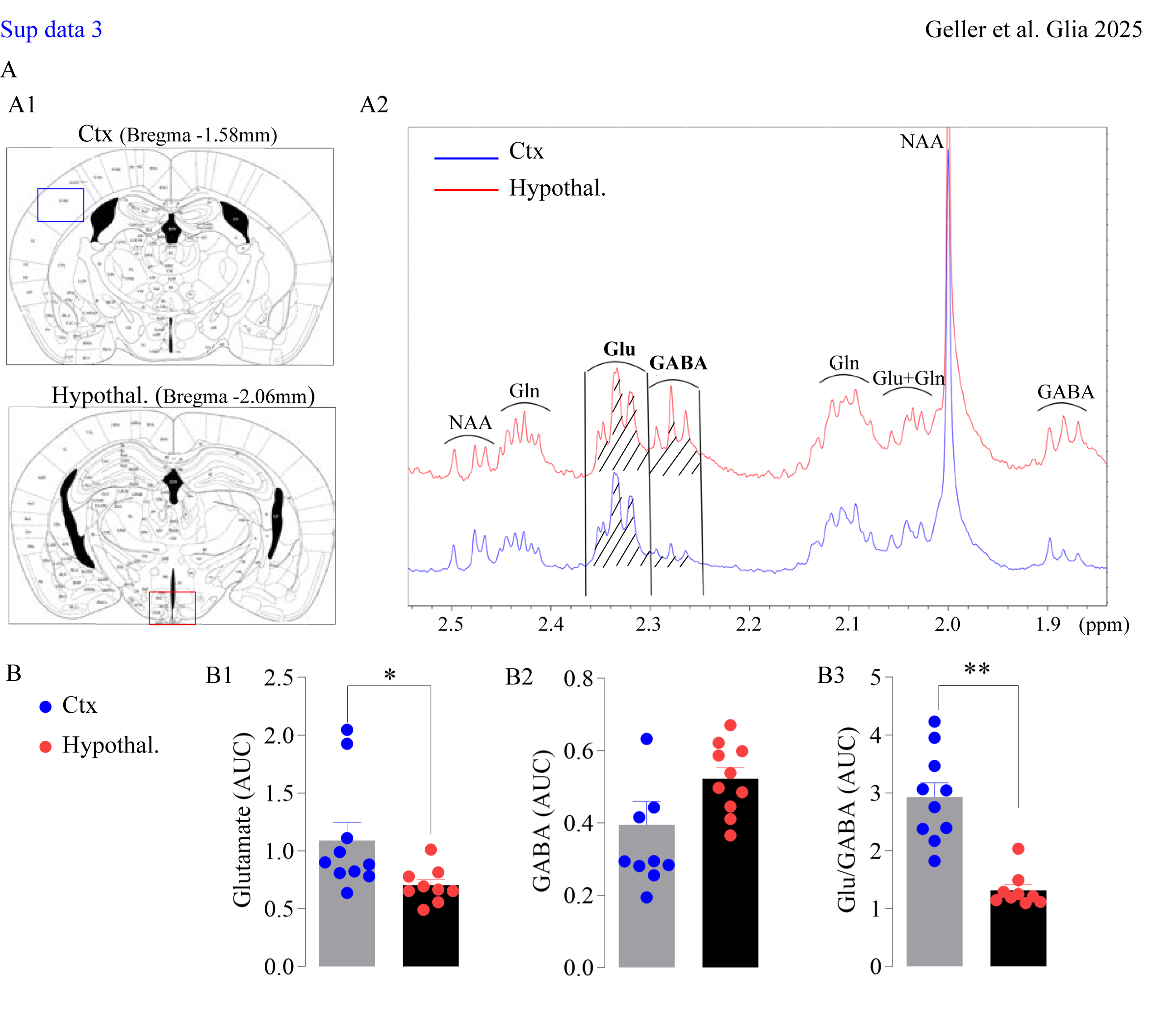

Supplement: Supplementary file 3 — Figure S3. Glutamate and GABA content in the barrel cortex and medio‐basal hypothalamus of adult male rats using HR‐MAS NMR spectroscopy. A. Schematic brain sections illustrating brain biopsies analyzed by HR‐MAS 1H‐NMR spectroscopy (A1) and representative NMR spectra of barrel cortex (blue line) and medio‐basal hypothalamus (red line) obtained from the same rat (A2). B. Comparative analysis of glutamate content (Glu, B1), GABA content (B2), and glutamate/GABA ratio (B3) between the barrel cortex (gray bar, blue circle) and the medio‐basal hypothalamus (black bar, red circle) obtained from the same rats (N = 9). Statistical analysis was performed using Wilcoxon test. *p < 0.05; **p < 0.01. Values indicate means ± SEM. [file GLIA-73-2253-s003.tif]

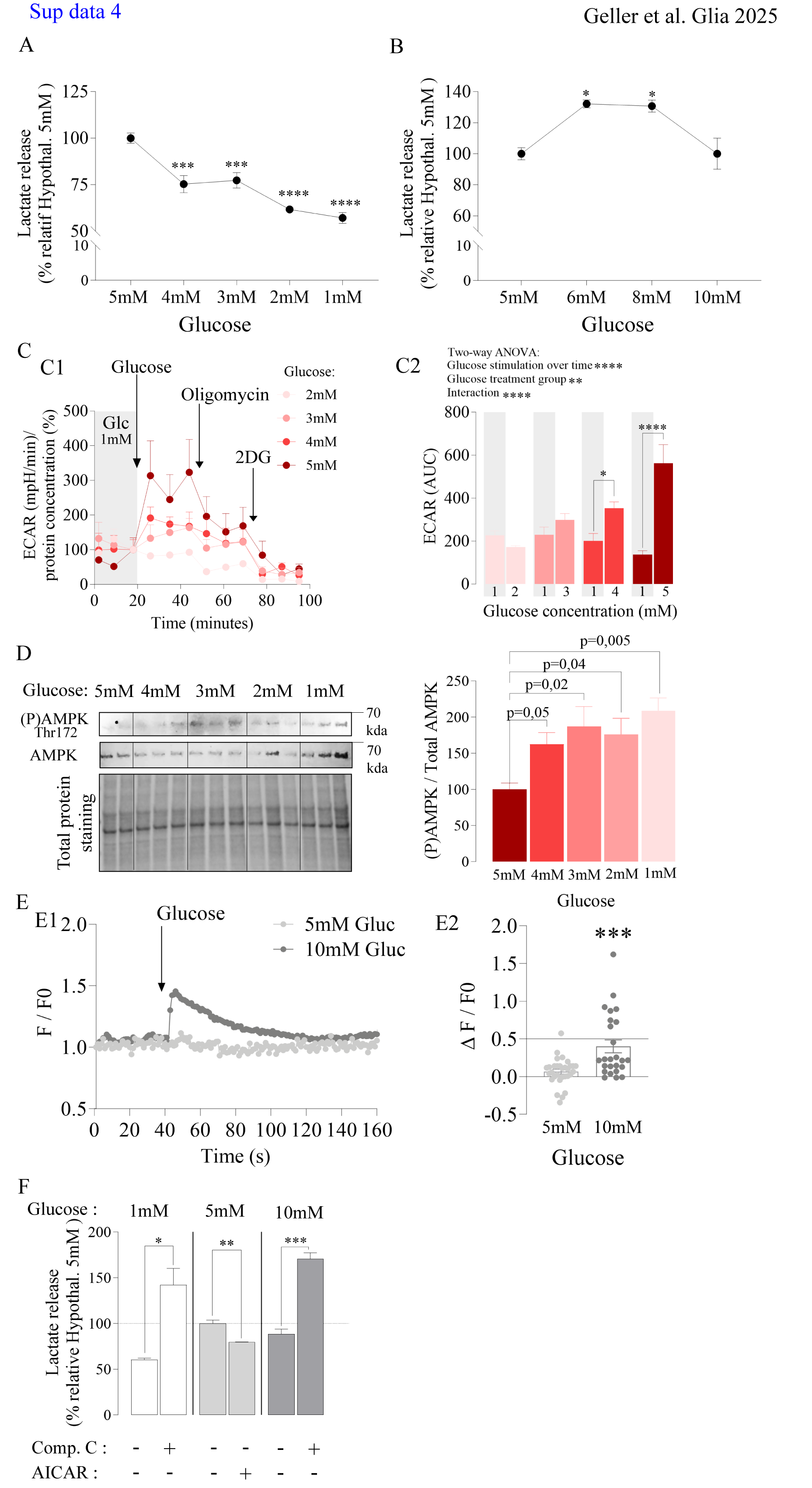

Supplement: Supplementary file 4 — Figure S4. Effects of glucose concentration variations on lactate release, calcium levels, and AMPK, as well as ACC phosphorylation in mouse hypothalamic astrocytes. A–B. Lactate release by hypothalamic astrocytes after exposure to different extracellular glucose concentrations from 5 to 1 mM (A, N = 2, n = 3) and from 5 to 10 mM (B, N = 1, n = 4–6). C. ECAR profiles of mouse astrocytes from hypothalamus at 1 mM glucose (gray background) and after adding glucose to a final concentration of 2, 3, 4, and 5 mM (N = 1, n = 5 per condition), oligomycin (1 μM) and 2DG (50 mM) (C1) and AUC of ECAR measurements hypothalamic astrocytes with 1 mM glucose (gray background) and after adding glucose (C2). D. Representative western blots and quantification of phosphorylated AMPK/total AMPK ratios of mouse hypothalamic astrocytes (from C57B6J pups) after extracellular glucose concentration variations (N = 3, n = 2–3). E. (E1) Representative graph of cytosolic Ca2+ transients in hypothalamic astrocytes stimulated with 5 or 10 mM glucose. (E2) Quantification of cytosolic Ca2+increase in hypothalamic astrocytes in response to 5 mM (pale gray, N = 5, n = 5–13 cells) or 10 mM (dark gray, N = 4, n = 5–14 cells) glucose stimulation. F. Lactate release by mouse hypothalamic astrocytes after extracellular glucose concentration variations in the presence or absence of Compound C (Comp. C, 1 mM) or AICAR (5 mM) (N = 1, n = 3). Statistical analysis was performed using unpaired t‐test or Mann–Whitney test for simple comparison and one‐way ANOVA followed by Dunnett’s multiple‐comparisons test with the control. *p < 0.05; **p < 0.01; ***p < 0.001. Values indicate means ± SEM. [file GLIA-73-2253-s004.tif]

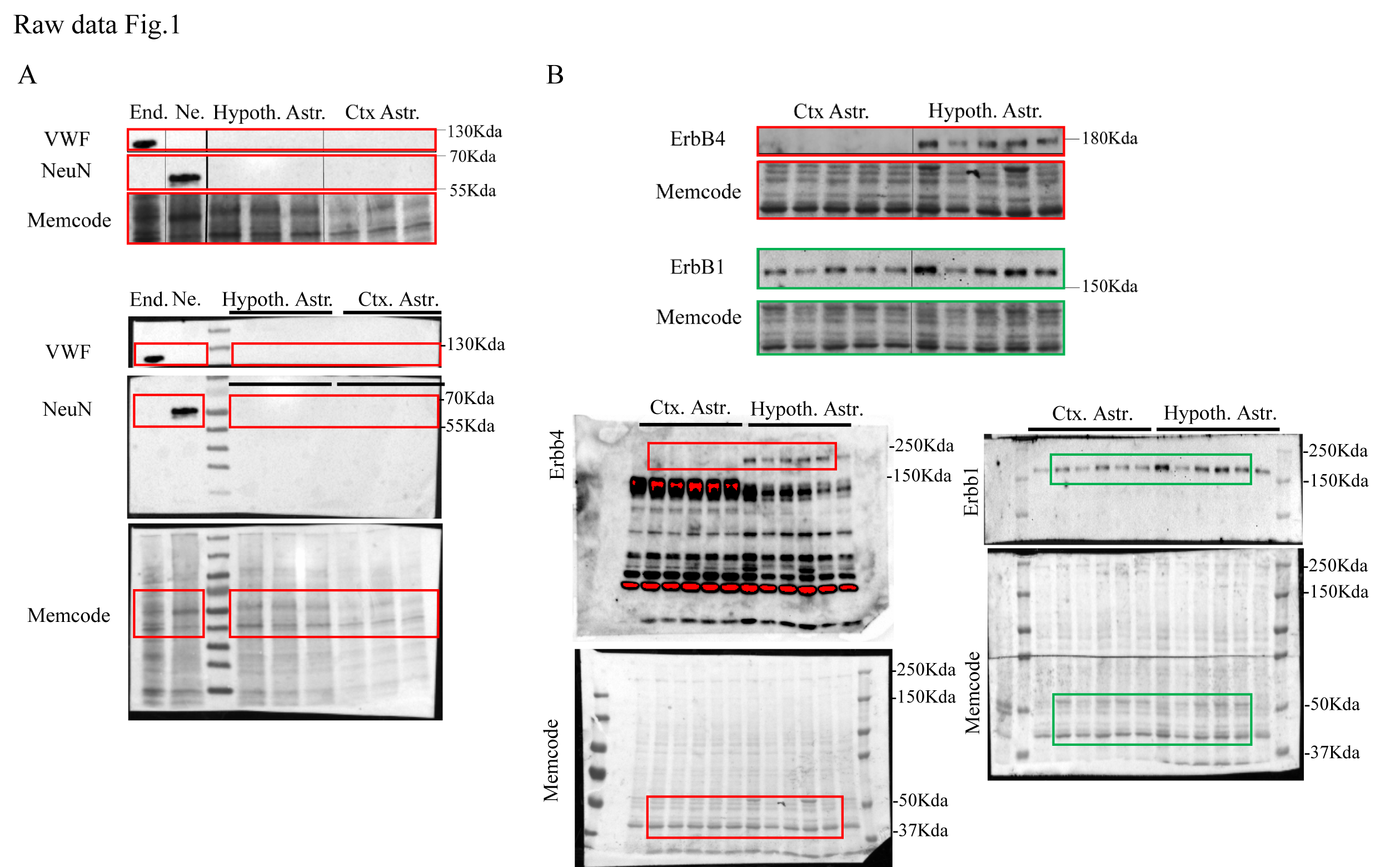

Supplement: Supplementary file 5 — Data S1. [file GLIA-73-2253-s002.zip › GLIA_70066_f1_RawDataFig1.tif]

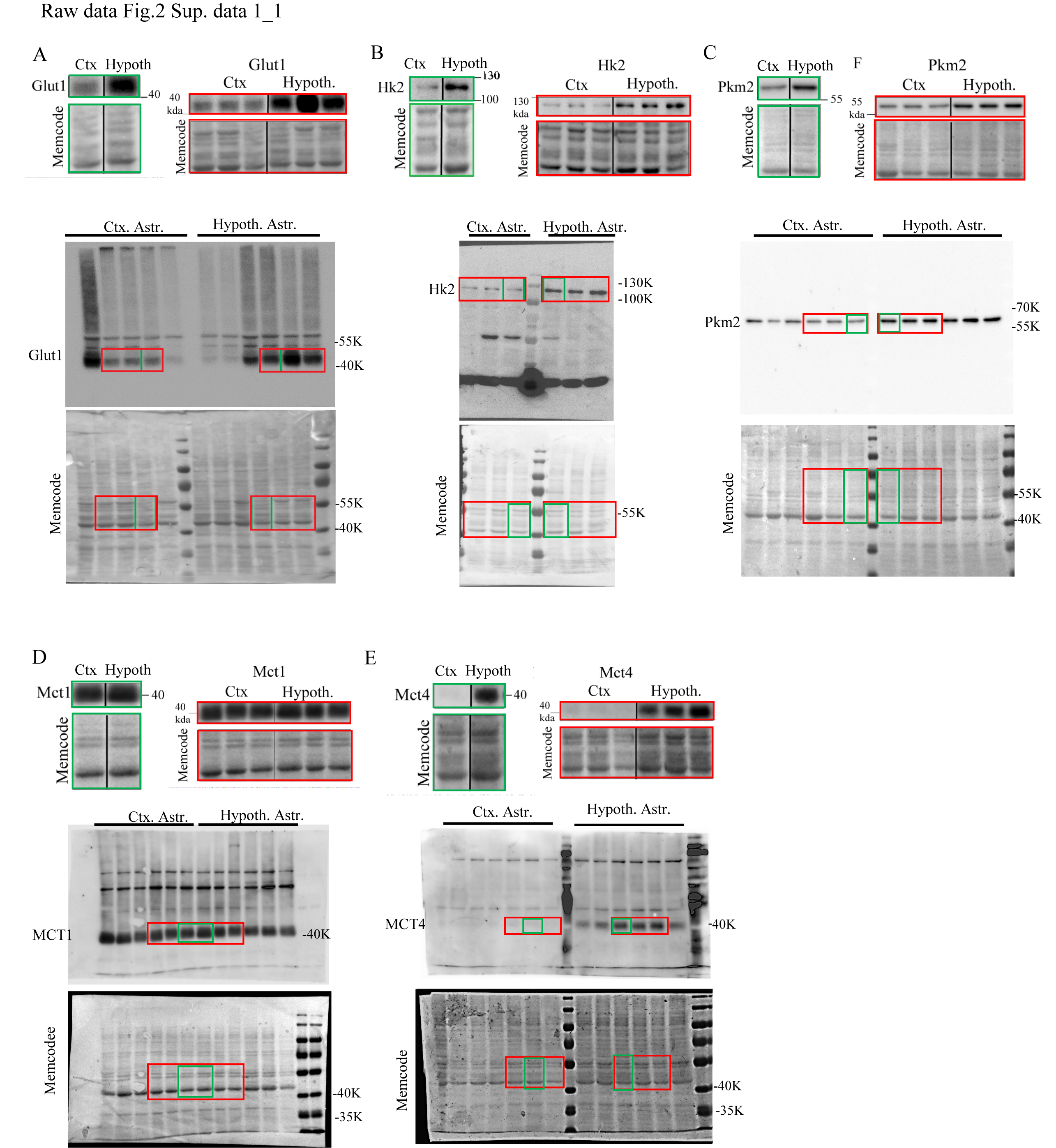

Supplement: Supplementary file 5 — Data S1. [file GLIA-73-2253-s002.zip › GLIA_70066_f2_RawDataFig2 Supdata1.tif]

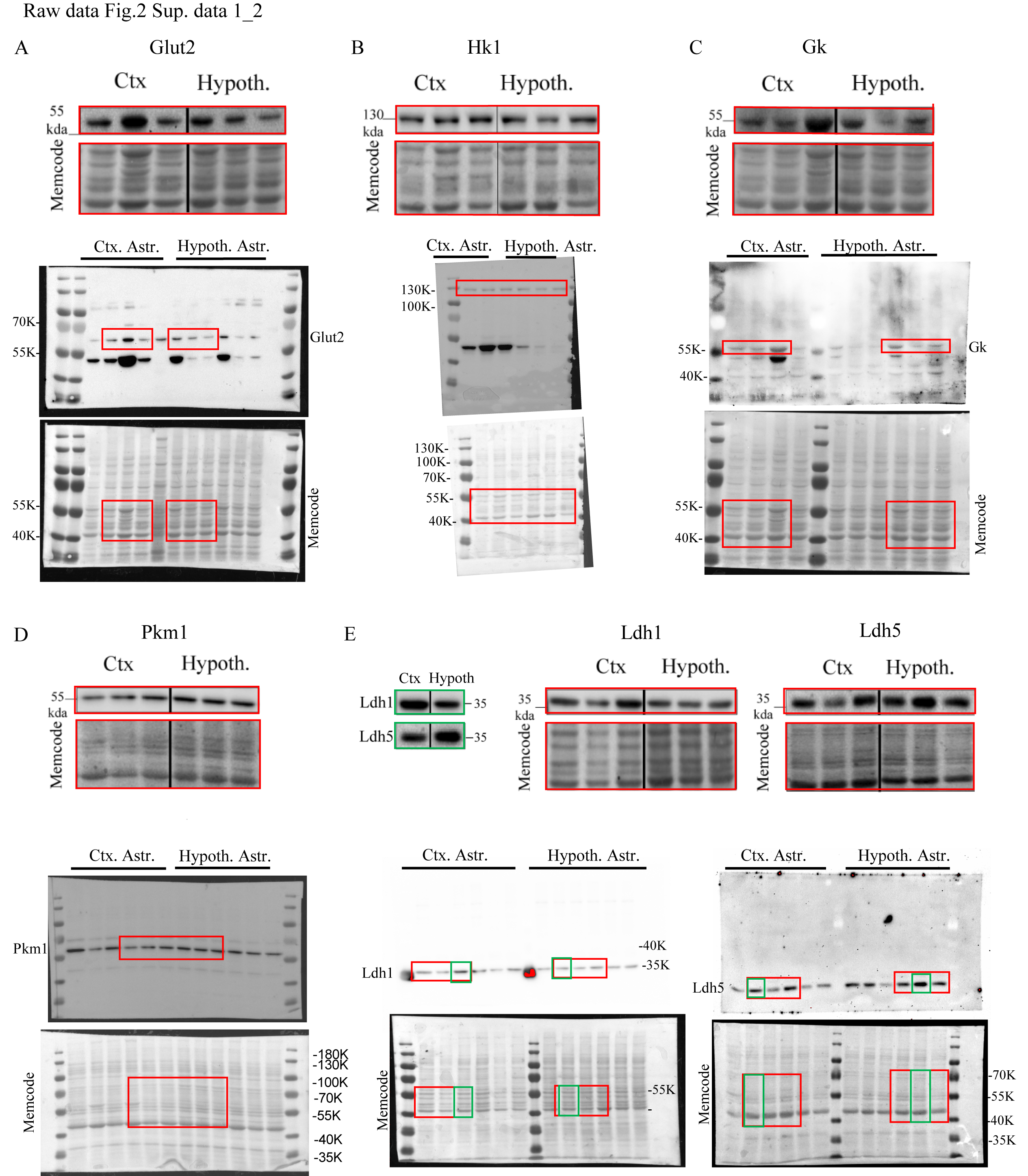

Supplement: Supplementary file 5 — Data S1. [file GLIA-73-2253-s002.zip › GLIA_70066_f2_RawDataFig2 Supdata1_2.tif]

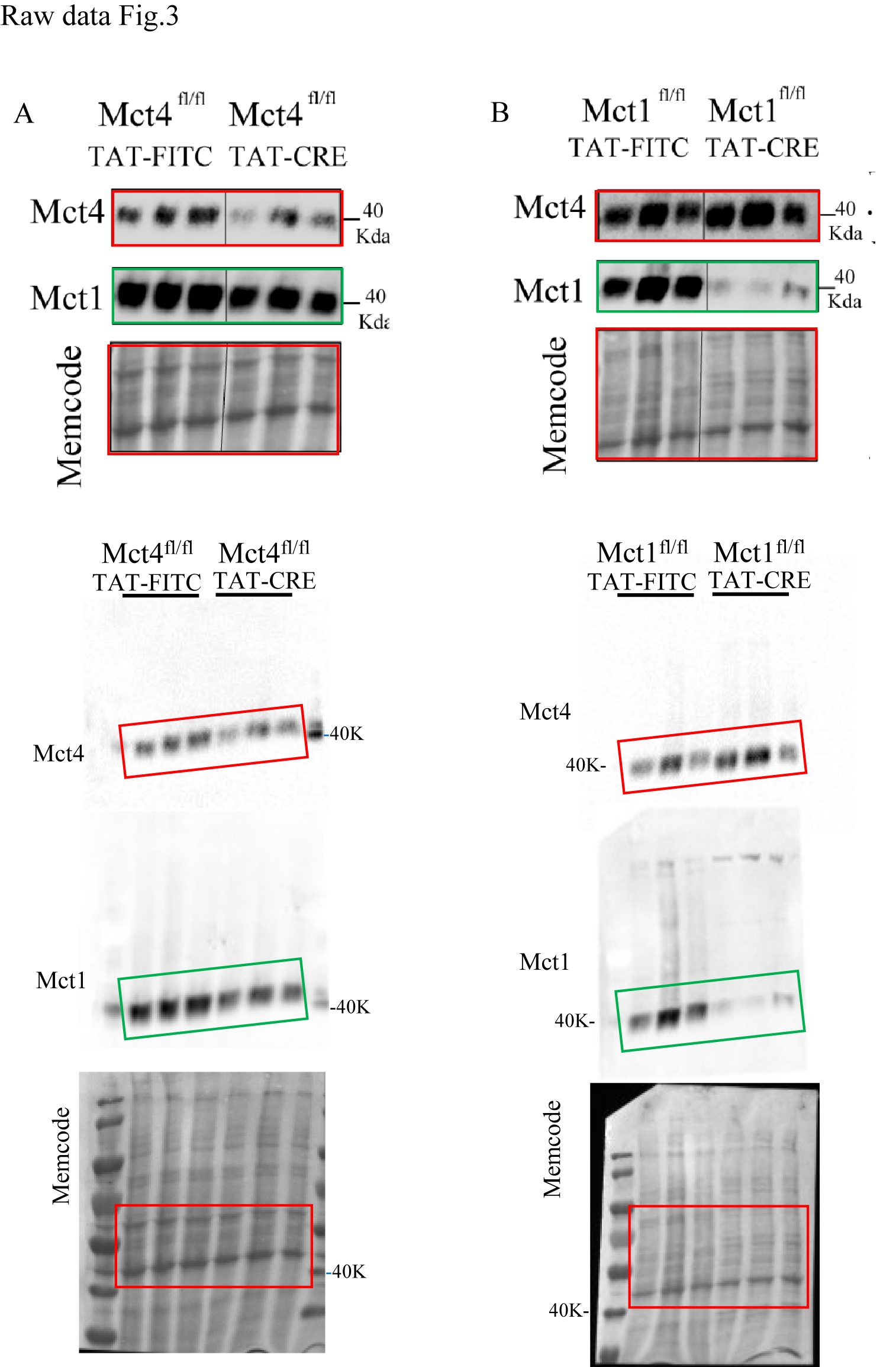

Supplement: Supplementary file 5 — Data S1. [file GLIA-73-2253-s002.zip › GLIA_70066_f3_RawDataFig3.tif]

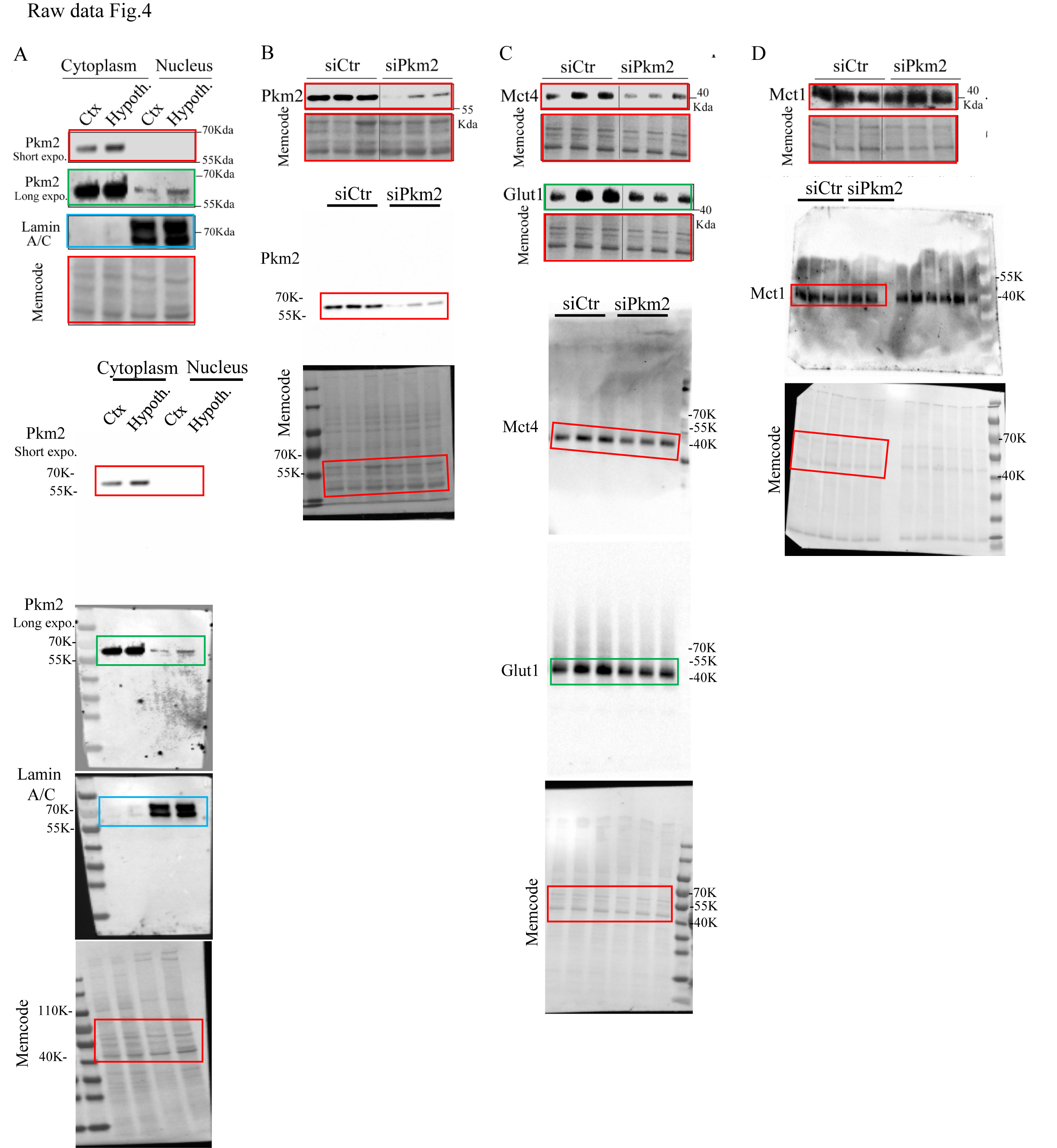

Supplement: Supplementary file 5 — Data S1. [file GLIA-73-2253-s002.zip › GLIA_70066_f4_RawDataFig4.tif]

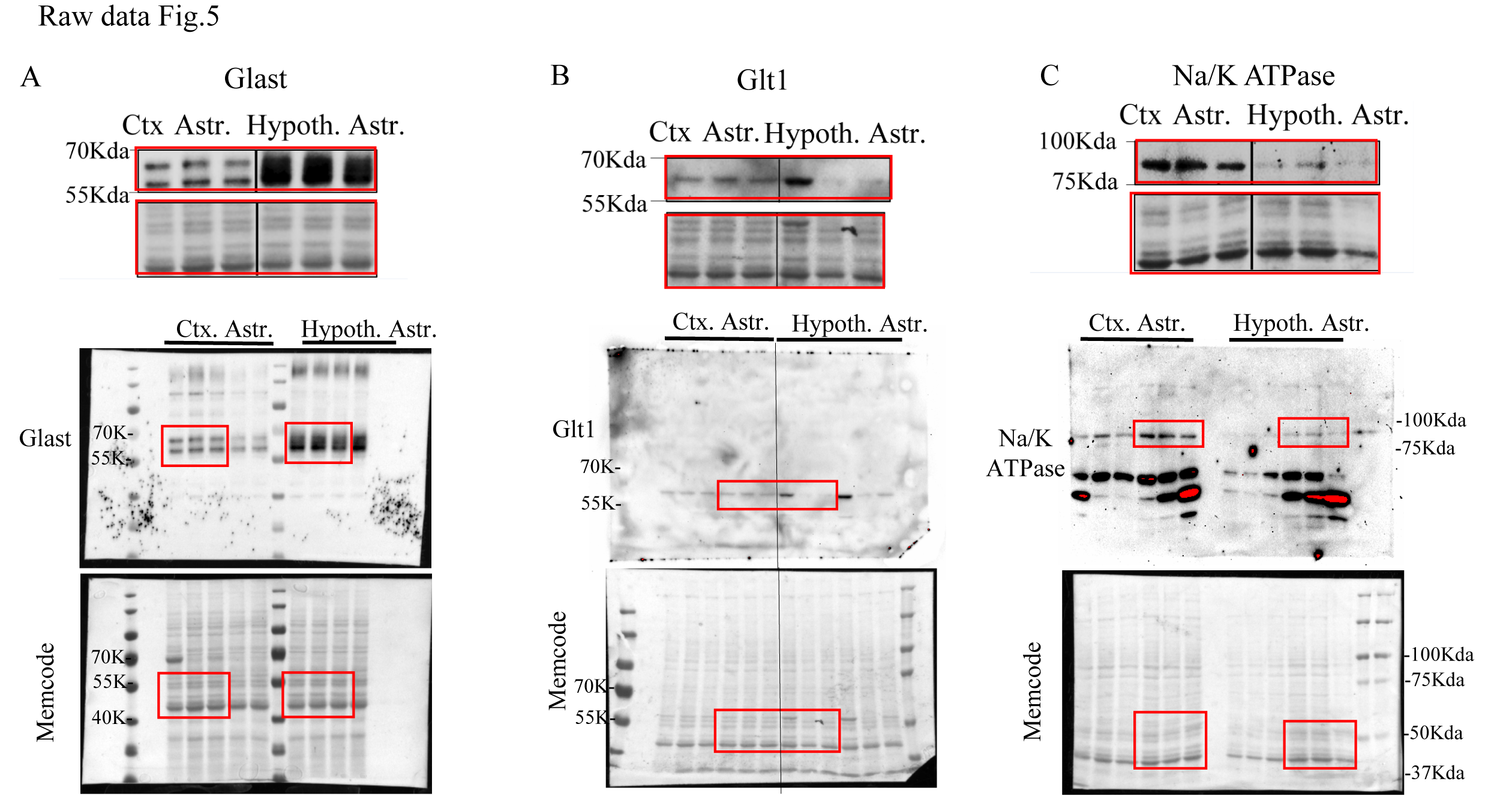

Supplement: Supplementary file 5 — Data S1. [file GLIA-73-2253-s002.zip › GLIA_70066_f5_RawDataFig5.tif]

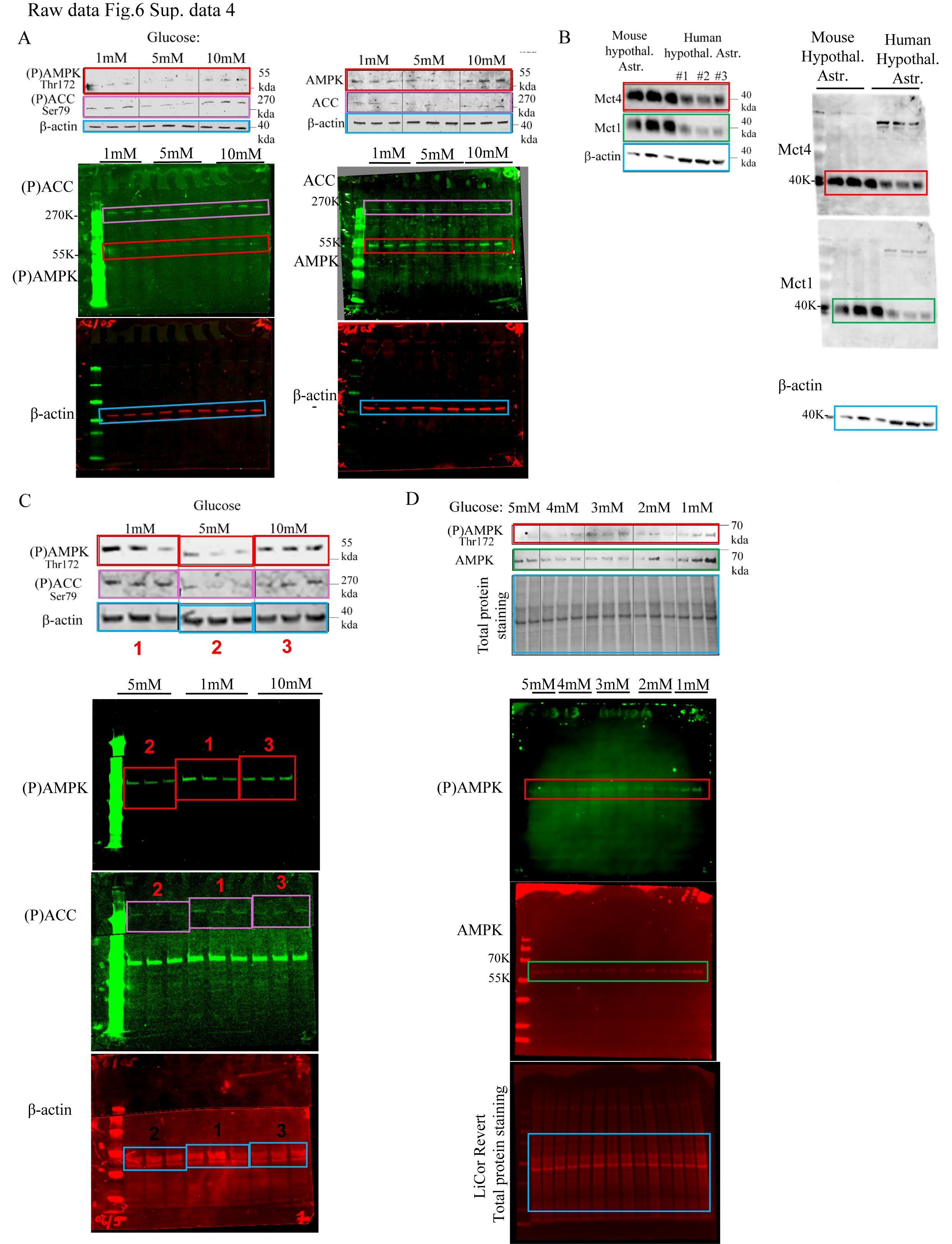

Supplement: Supplementary file 5 — Data S1. [file GLIA-73-2253-s002.zip › GLIA_70066_f6_RawDataFig6 Supdata4_2.tif]
